# Chromatin Remodeller BRD9 Orchestrates Odontoblastic Differentiation via Coordinating RUNX2‐KLF4

**DOI:** 10.1111/cpr.70269

**Published:** 2026-08-06

**Authors:** Wenrui Zeng, Yuxiu Lin, Yongyan Gao, Delan Huang, Yuanyuan Li, Honglei Ruan, Guohua Yuan, Huan Liu, Zhi Chen

**Affiliations:** ^1^ State Key Laboratory of Oral & Maxillofacial Reconstruction and Regeneration, Key Laboratory of Oral Biomedicine Ministry of Education, Hubei Key Laboratory of Stomatology, School & Hospital of Stomatology Wuhan University Wuhan China; ^2^ Department of Cariology and Endodontics, School of Stomatology Wuhan University Wuhan China; ^3^ Xiamen Key Laboratory of Stomatological Disease Diagnosis and Treatment Stomatological Hospital of Xiamen Medical College Xiamen China; ^4^ Frontier Science Center for Immunology and Metabolism Wuhan University Wuhan China; ^5^ Hubei Provincial Key Laboratory of Developmentally Originated Disease Wuhan China; ^6^ TaiKang Center for Life and Medical Sciences Wuhan University Wuhan China

**Keywords:** cell differentiation, chromatin remodelling, epigenomics, odontoblasts, odontogenesis, transcription factors

## Abstract

Dentinogenesis, a process essential for tooth function, relies on the precise differentiation of dental papilla cells into odontoblasts. This lineage commitment is governed by complex transcription factor networks. RUNX2 and KLF4, known key TFs co‐expressed in this process, were proven to operate synergistically, but the specific cofactors coordinating their activity at the chromatin level remain unknown. Here, we identify the chromatin remodeller BRD9 as a novel interactor of both RUNX2 and KLF4 during odontoblastic differentiation. To probe their shared function, we generated neural crest‐specific conditional knockout mice. *Wnt1*‐Cre; *Brd9* ^fl/fl^ mice exhibited a disordered odontoblast layer with reduced secretion of extracellular matrix proteins (DMP1 and DSPP), strikingly phenocopying the compound *Wnt1*‐Cre; *Runx2* ^fl/wt^; *Klf4* ^fl/fl^ mutants. In vitro, chemical BRD9 degradation suppressed odontoblastic differentiation and mineral deposition. Integrative analyses of RNA‐seq and ATAC‐seq revealed that BRD9 maintained chromatin accessibility at odontogenesis‐associated regions enriched with RUNX2 and KLF4 motifs, specifically at the *Fam20c* enhancer, thereby facilitating RUNX2 and KLF4 binding for transcriptional activation. Crucially, exogenous supplementation of FAM20C protein partially rescued the odontoblastic differentiation defects upon BRD9 loss. These findings establish BRD9 as a critical epigenetic orchestrator that coordinates RUNX2‐KLF4 synergy to activate key odontogenic genes like *Fam20c*, thus affecting odontoblastic differentiation and dentinogenesis.

## Graphical abstract

During odontoblast lineage commitment, the chromatin remodeller BRD9 acts as a critical epigenetic coordinator, orchestrating the chromatin landscape to facilitate synergistic binding of key transcription factors RUNX2 and KLF4 to target loci for odontogenesis. Loss of BRD9 disrupts the precise odontoblastic differentiation program, resulting in defective dentinogenesis.

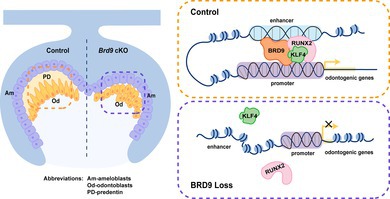

## 1 Introduction

Tooth morphogenesis is typically orchestrated through a sequence of spatiotemporal molecular crosstalk between the epithelial and mesenchymal compartments [1]. During the cap‐to‐bell transition, cranial neural crest (CNC)‐derived dental mesenchymal cells give rise to the dental papilla and subsequently differentiate into odontoblasts—a terminally differentiated population that secretes organic matrix components to form mineralised dentine [2, 3]. This odontoblastic differentiation, fundamental to dentinogenesis, is precisely regulated by intricate transcription factor (TF) networks and characterised by the expression of specific markers including DSPP (dentine sialophosphoprotein) and DMP1 (dentine matrix protein 1) [4].

Transcriptional regulation responsible for determining odontoblast lineage commitment is inevitably accompanied by dynamic alterations in chromatin accessibility [5, 6, 7, 8, 9]. Among critical TFs, RUNX2 (runt‐related transcription factor 2) and KLF4 (kruppel‐like factor 4) act synergistically in promoting DSPP and DMP1 expression [10, 11, 12] and activating the TGF‐β signalling pathway [13]. Notably, KLF4‐dependent accessible chromatin regions are enriched with RUNX motif [13], suggesting a cooperative mechanism at the chromatin level. However, the molecular basis underlying their transcriptional synergy remains unclear. Specifically, how the chromatin remodeller coordinates RUNX2 and KLF4 to modulate odontoblastic differentiation transcription has yet to be elucidated.

Chromatin remodelling, a prominent epigenetic mechanism, alters genomic DNA accessibility for key genetic processes including transcription [14]. Recent studies have highlighted the spatiotemporal functions of chromatin remodellers during mammalian embryonic multilineage commitment [15] and craniofacial development [16]. BRD9 (bromodomain containing 9), a core subunit exclusive to the ncBAF (non‐canonical BRG1/BRM‐associated factor) chromatin remodelling complex [17], contains a bromodomain that acts as a histone acetylation ’reader’ and contributes to an accessible chromatin state to orchestrate gene regulation [18]. In embryonic stem cells, BRD9, as part of BAF complex, cooperates with pluripotent TFs (e.g., KLF4, SP5) [19] and interacts with multiple factors (e.g., BRD4, SMAD2/3, β‐CATENIN and P300) [20] in regulating pluripotency programmes and signalling pathways, thus affecting cell self‐renewal and differentiation. These suggest that BRD9 facilitates the recruitment and assembly of transcriptional machinery to specific loci.

Emerging evidence has demonstrated that BRD9 is indispensable for osteogenesis in both mouse and zebrafish models. It modulates osteoblast differentiation by collaborating with β‐catenin to regulate *Sp7* transcription [21]. Given the well‐recognised parallels between osteogenesis and dentinogenesis—the shared developmental origins from neural crest, similar transcriptional regulatory networks and matrix secretion, these findings suggest the analogous potential of BRD9 in dentinogenesis. Nevertheless, little evidence exists regarding the necessary role of chromatin remodellers like BRD9 in odontoblastic differentiation and whether BRD9 interacts with RUNX2‐KLF4 to mechanistically regulate this process remains elusive.

In this present study, we found a striking overlap between binding proteins of RUNX2 and KLF4 which includes the chromatin remodeller BRD9. Then, we demonstrated that BRD9 is an essential cofactor of RUNX2‐KLF4 complex in orchestrating odontoblastic differentiation in vivo and in vitro. Neural crest‐specific ablation of *Brd9* resulted in disorganised odontoblast morphology and impaired dentine matrix formation, a phenotype closely recapitulated by compound *Runx2* and *Klf4* mutants. Notably, while single deletion of *Runx2* or *Klf4* produced relatively mild defects, their joint loss led to exacerbated odontoblastic abnormalities, underscoring their synergistic requirement in dentinogenesis and phenocopying *Brd9* loss. Complementary in vitro investigations with chemical BRD9 degradation further confirmed its critical role, as evidenced by impaired expression of odontoblastic markers, attenuated mineralisation and global transcriptional alterations. Integrated transcriptomic and epigenomic analyses revealed that BRD9 maintains chromatin accessibility at regions enriched with RUNX2 and KLF4 motifs, thereby facilitating their cooperative binding to specific odontogenic loci including the *Fam20c* enhancer. Collectively, these findings establish BRD9 as a pivotal epigenetic coordinator that enables synergistic transcription factor activity to drive odontoblast lineage commitment, providing mechanistic insight into chromatin‐mediated regulation of dentinogenesis and highlighting potential epigenetic targets for dentine regeneration.

## 2 Materials and Methods

### 2.1 Animals and Sample Collection

All animal studies conformed to the ARRIVE guidelines and were approved by the Institutional Animal Care and Use Committees at School and Hospital of Stomatology of Wuhan University (No. S07924120J). Brd9fl/fl mice (Strain NO. T008489) were purchased from GemPharmatech Co. Ltd. (Nanjing, China). Runx2fl/wt mice (Strain S‐CKO‐01564) were purchased from Cyagen Biosciences Inc. (Suzhou, China). Wnt1‐Cre mice (Strain #003829) were obtained from the Jackson Laboratory, while Klf4fl/fl mice (Stock NO. 029877‐MU) were purchased from the Mutant Mouse Regional Resource Centers (MMRRC) as previously described [13]. Conditional knockout mice on the C57BL/6J background (Wnt1‐Cre; Brd9fl/fl and Wnt1‐Cre; Runx2fl/wt; Klf4fl/fl) were generated to observe dental phenotypes. Mouse heads were carefully dissected and fixed in 4% paraformaldehyde (PFA) overnight and then, decalcified in 10% ethylenediaminetetraacetic acid (EDTA, pH 7.4), dehydrated and embedded in paraffin for histological analysis. Both male and female embryos or pups were used in this study and littermate controls were used for all experiments and analysis.

### 2.2 Cell Culture and Reagents

The primary mouse dental papilla cells (mDPCs) were isolated from first molars of E16.5 wildtype embryos, digested with 0.25% trypsin for 15 min and then seeded. To overcome their short lifespan, the immortalised cell line mDPC6T previously established was used for partial experiments as a stable cell model. This cell line was established from 1st lower molar dental papilla of E16.5 mouse embryos, using SV40 T antigen‐based immortalisation strategy, which maintained the differentiation capacity of primary cultured mDPCs [8, 22]. They were cultured in Dulbecco's modified Eagle's medium (DMEM, Hyclone) containing 10% foetal bovine serum (FBS, Hyclone) and 1% penicillin/streptomycin (P/S, Hyclone) in a humidified cell incubator at 37°C and 5% CO_2_. For odontoblastic differentiation induction, cells were supplemented with 50 mg/mL ascorbic acid (Sigma‐Aldrich), 10 mM sodium β‐glycerophosphate (Sigma‐Aldrich) and 10 nM dexamethasone (Sigma‐Aldrich). HEK293T cells were cultured in the same DMEM with 10% FBS. All media were refreshed every 2 days.

For degradation of BRD9, cells were treated with dBRD9‐A (Tocris, No. 6943), a highly selective BRD9‐specific PROTAC (proteolysis‐targeting chimera) [23, 24]. This reagent was dissolved by DMSO and DMSO alone served as the negative control. To observe the function of FAM20C, recombinant mouse FAM20C protein (rmFAM20C, TargetMOI, TMPH‐02647) was reintroduced in a rescue experiment at the concentration of 1000 ng/mL.

### 2.3 Co‐Immunoprecipitation and Mass Spectrometry Analysis (Co‐IP/MS)

Co‐IP was performed following a standard protocol. mDPC6T cells without odontoblastic induction were lysed with NP40 lysis buffer (Beyotime). Lysates were precleared with a control rabbit IgG antibody (ABclonal, AC005) under incubation on ice for 1 h. Then, pre‐washed protein A/G magnetic beads (Selleck, B23202) were added and rotated at 4°C for 30 min to remove non‐specifically binding proteins. Supernatants were finally collected using magnetic stand and were incubated with IgG control, anti‐RUNX2 (Cell Signalling Technology, D1L7F) or anti‐KLF4 (Abcam, ab214666) antibodies, with gentle rotation overnight at 4°C. The next day, protein A/G magnetic beads were added and incubated on the rotator at 4°C for 4 h. Beads binding with antibody‐selected proteins were collected by centrifugation at 6400 rpm for 5 min. After washing 4 times, loading buffer was added to the washed beads to elute the immunoprecipitated proteins. The mixtures (IgG control, IP‐RUNX2 and IP‐KLF4; each with two biological replicates) were submitted to BGI Genomics Co. Ltd. (Shenzhen, China) for mass spectrometry analysis. Data were searched using Mascot [25] engine (v2.3.02) and filtered with Percolator [26] (PSM‐level FDR ≤ 0.01). Label‐free quantification was done using the iBAQ algorithm [27].

### 2.4 Haematoxylin and Eosin (H&E) Staining

Paraffin samples were subjected to sagittal sectioning of 6‐μm thickness. After deparaffinisation and rehydration steps, haematoxylin and eosin staining (Beyotime) was performed following a standard procedure.

### 2.5 Morphometric Analysis

The cusp height was defined as the perpendicular distance from the highest cusp tip to the cervical loop reference line (connecting the lowest visible points of the buccal and lingual cervical loops) in sections.

The depth of invagination was defined as the perpendicular distance from the deepest point of the central epithelial invagination (sulcus base) to the imaginary line connecting the tips of two adjacent cusps [28].

At PN0.5, the pale pink, homogeneous tissue between the odontoblast layer and the inner enamel epithelium was identified as predentin, which reflected the matrix secretion capacity of newly differentiated odontoblasts. Predentin thickness was measured as the perpendicular distance from the odontoblastic cell layer to the distal boundary of the pale‐staining matrix zone (or to the ameloblastic cell layer when it was not yet apparent). The values of the thickest regions of predentin layer in the cusps were used for statistics.

All measurements were performed on the most representative coronal sections from a series of consecutive sections, defined as the sections displaying the largest and most characteristic tooth germ morphology. To minimise the potential variation in sectioning plane, we used the ratios of epithelial invagination depth to cusp height and predentin thickness to cusp height as normalised indices.

### 2.6 Immunohistochemistry (IHC)

The sections were dewaxed, rehydrated and boiled in 10 mM/L citrate buffer (pH 6.0) for antigen retrieval. The slides were blocked and incubated with anti‐BRD9 (Abcam, ab259839, 1:100), anti‐RUNX2 (Cell Signalling Technology, D1L7F, 1:400) or anti‐KLF4 (Abcam, ab214666, 1:100) antibodies at 4°C overnight. After incubating with a horseradish peroxidase (HRP) secondary antibody, slides were stained using the diaminobenzidine reagent kit (Maixin) and counterstained by haematoxylin. Negative controls were obtained by incubating with PBS as replacement of first antibody.

### 2.7 Immunofluorescence (IF)

After dewaxing, rehydration and antigen retrieval, paraffin sections were blocked with 2.5% bovine serum albumin (BSA, Sigma–Aldrich) for 1 h at 37°C and incubated with primary antibodies against DMP1 (BioVision, 3844, 1:100), DSPP (ABclonal, A8413, 1:100), SIX2 (Proteintech, 11562‐1‐AP, 1:100), PAX9 (Santa Cruz, sc‐56823, 1:200), MSX1 (R&D Systems, AF5045, 1:400), Ki67 (Cell Signalling Technology, D3B5, 1:200), Cleaved Caspase‐3 (CC‐3, Cell Signalling Technology, Asp175, 1:200), RUNX2 (Cell Signalling Technology, D1L7F, 1:200), KLF4 (Abcam, ab214666, 1:200) or FAM20C (Proteintech, 25395‐1‐AP, 1:100) at 4°C overnight. After washing with PBS three times, they were incubated with Alexa Fluor 594/488 secondary antibody (AntGene, ANT049/ANT045) for 1 h at 37°C.

For cell double immunofluorescence staining, cells were seeded on chamber slides, fixed with 4% PFA for 15 min, permeabilised with 0.1% Triton X‐100 for 10 min and blocked. They were first stained with anti‐BRD9 or anti‐KLF4 antibodies at 4°C overnight, followed by Alexa Fluor 594 secondary antibody. Then, after antibody stripping with mIHC antibody stripping buffer (Recordbio) for 15 min twice and re‐blocking, cells were stained again with anti‐RUNX2 or anti‐KLF4 antibodies at 37°C for 1 h and incubated with Alexa Fluor 488 secondary antibody.

All samples were mounted with an antifade mounting medium containing DAPI (Beyotime) for nuclear counterstaining and were photographed under a fluorescence microscope (Zeiss).

### 2.8 Proximal Ligation Assay (PLA)

mDPC6T cells were seeded on a chamber slide and fixed with 4% PFA. After permeabilisation, PLA experiments were performed according to the instructions of the Duolink In Situ Red Starter Kit Mouse/Rabbit (Sigma‐Aldrich). After blocking with the blocking solution for 1 h at 37°C, the cells were incubated with both rabbit and mouse antibodies simultaneously overnight at 4°C and then incubated with a pair of PLA probes (Donkey anti‐Rabbit IgG plus and Donkey anti‐Mouse IgG minus) and subjected to ligation and amplification. Duolink In Situ Mounting Medium with DAPI (Sigma‐Aldrich) was used to mount the slides with a cover slip. The red fluorescent spots (λex = 594 nm) indicated the occurrence of protein–protein interactions.

### 2.9 Western Blot Analysis

Cells were lysed using lysis buffer containing a protease inhibitor cocktail. Total protein supernatants were extracted after sonication and centrifugation. Protein concentrations were measured and standardised using a BCA Protein Assay Kit (Thermo Fisher Scientific) and were resuspended in 5× SDS‐PAGE (Biosharp) loading buffer at 95°C for 10 min. Equal amounts of proteins were loaded and separated via 10% polyacrylamide gel electrophoresis (Vazyme) and then transferred onto polyvinylidene fluoride (PVDF) membranes (Merck Millipore). The membranes were blocked with rapid protein free blocking buffer (Pumoke) and subsequently incubated at 4°C overnight with following primary antibodies: anti‐BRD9 (Abcam, ab259839, 1:1000), anti‐RUNX2 (Cell Signalling Technology, D1L7F, 1:1000), anti‐KLF4 (Abcam, ab214666, 1:1000), anti‐DMP1 (BioVision, 3844, 1:1000), anti‐DSPP (ABclonal, A8413, 1:1000), anti‐FAM20C (Proteintech, 25395‐1‐AP, 1:1000), anti‐β‐ACTIN‐HRP conjugated (Pumoke, PMK058, 1:5000). The next day, membranes were incubated with HRP‐conjugated IgG secondary antibodies (Merck Millipore, AP132P, 1:4000) for 1 h at room temperature. Signals were detected by WesternBright ECL solution (Advansta) under an enhanced chemiluminescence system. ImageJ software was used to quantify the density of targeted protein bands.

### 2.10 RNA Extraction and Quantitative Real‐Time PCR (qRT‐PCR)

Total RNA was extracted using the FastPure Cell/Tissue Total RNA Isolation Kit (Vazyme), reverse‐transcribed into cDNA using 5 × HiScriptII qRT SuperMix (Vazyme) and analysed by qRT‐PCR using ChamQ SYBR qPCR Master Mix (Vazyme) under the CFX Connect Real‐Time System. For quantification, the ΔCt method was used to calculate relative changes and *Gapdh* was used as the internal normalisation control. Sequences of primers used in qRT‐PCR were listed in Table S1.

### 2.11 Cell Counting Kit‐8 (CCK‐8)

Cell viability was analysed by the cell counting kit‐8 (CCK‐8, Beyotime). Cells were seeded in 96‐well plates and treated with dBRD9‐A or DMSO. We measured the optical density values of the culture after 1 h of 10% CCK‐8 solution incubation at 0–5 days respectively using a microplate spectrophotometer (Bio‐Tek).

### 2.12 Alizarin Red Staining

Differentiation induced mDPCs were fixed by 4% PFA, washed and then stained in 1% Alizarin red S (ARS, Sigma‐Aldrich) solution (pH 4.2) for 10 min at room temperature. After washing with PBS to remove unbound dye, cells were finally photographed under the inverted microscope (Zeiss). For semi‐quantification, 10% cetylpyridinium chloride (CPC, Sigma‐Aldrich) buffer (pH 7.0) was added to each well and incubated for 1 h on the platform shaker at room temperature. Absorbance was measured at 562 nm. Three independent experiments using distinct cell litters were done and technical replicates (duplicate wells) were averaged for statistical analysis.

### 2.13 RNA Sequencing (RNA‐seq)

E16.5 mDPCs were treated with dBRD9‐A or DMSO under odontoblastic induction for 5 days. Then, RNA was extracted using Trizol reagent (Ambion) and sent to the company Novogene Co. Ltd. (Beijing, China) for RNA‐seq library preparation and analysis. RNA‐seq was performed on three biologically independent replicates per group. Kallisto (v.0.44.0.) [29] was used to read counts for quantifying the transcripts and Sleuth R package [30] was used for differential analysis. The R software was used to draw the volcano plot and perform gene ontology (GO), Kyoto Encyclopaedia of Genes and Genomes (KEGG) enrichment analysis and GSEA (Gene Set Enrichment Analysis).

### 2.14 Assay for Transposase‐Accessible Chromatin With High‐Throughput Sequencing (ATAC‐seq)

After odontoblastic induction for 5 days, mDPCs were harvested and 50,000 cells per replicate were collected for ATAC‐seq library generation with the Hyperactive ATAC‐seq Library Prep Kit (Vazyme, TD711). Cells were washed by TW buffer twice, resuspended in lysis buffer and centrifuged to pellet nuclei, which were immediately submitted to tagmentation mix with Tn5 transposase (TTE Mix V50) at 37°C for 30 min. Then, DNA was extracted and purified using ATAC DNA Extract Beads and eluted in nuclease‐free ddH2O, followed by amplification and indexing with TruePrep Index Kit V4 for Illumina (Vazyme, TD204). Finally, all libraries were purified with ATAC DNA Clean Beads and sent to sequence at Annoroad Gene Technology Co. Ltd. (Beijing, China). Libraries were constructed from two biologically independent replicates for the normal control (NC) group and three for BRD9 degradation (dB) group. After trimming and mapping by trimmomatic v.0.38 [31] and bowtie2 [32], PCR duplicates were removed using Picardtools. Bigwig files were generated by Deeptools2 [33] and MACS2 [34] was applied for peak calling. DiffBind [35] was used to identify the differentially accessible nucleosome‐free regions (NFRs), while HOMER was used to identify the motifs, annotate the differentially accessible regions (DARs) and predict target regions. Data visualisation was performed on the UCSC Genome Browser.

### 2.15 Plasmid Construction

The promoter region of *Fam20c* and an intergenic DAR (chr5: 138730873–138,731,272) putative as the enhancer were PCR‐amplified using the genomic DNA extracted from mDPCs as the template. The synthetic fragments were cloned into the pGL3‐basic plasmid through in‐fusion cloning for 2 constructs: the pGL3‐Promoter (Pro) plasmid and pGL3‐Promoter‐Enhancer (Pro‐Enh) plasmid. Then, site‐directed mutagenesis was performed to delete predicted RUNX2‐KLF4 binding motif sites. An inverse PCR strategy was adopted using Q5 High‐Fidelity DNA Polymerase (NEB), followed by seamless cloning using 2 × CE Mix (Vazyme) to generate the mutants (Pro‐Enh‐△motif1/2/3). All constructs were validated through sequencing. Sequences of insert regions and primers for construction were listed in Table S2.

### 2.16 Luciferase Reporter Assay

Using Lipofectamine 2000 reagent (Invitrogen), mDPC6T cells were co‐transfected with above plasmids to be tested and pRL‐TK plasmid for normalisation. Partial groups were treated with dBRD9‐A for 24 h after transfection. And 48 h later, cells were harvested and the luciferase expression activity was detected using Dual Luciferase Reporter Gene Assay Kit (Yeasen, 11402ES) under GloMax 20/20 Luminometer (Promega).

### 2.17 CUT&Tag (Cleavage Under Targets and Tagmentation)‐qPCR

After odontoblastic induction for 5 days, CUT&Tag assay was performed according to the manufacturer's instructions for Hyperactive Universal CUT&Tag Assay Kit (Vazyme, TD904) and qPCR was used to quantify the expression of specific DNA sites binded by RUNX2 and KLF4. Briefly, approximately 100,000 cells in each biological replicate were harvested and incubated with ConA Beads Pro at room temperature for 10 min. Then, bead‐bound cells were resuspended in precooled Antibody Buffer and incubated with primary antibody (1:100 dilution) against RUNX2 (Cell Signalling Technology, D1L7F), KLF4 (Proteintech, 11,880–1‐AP) or rabbit control IgG (ABclonal, AC005) overnight at 4°C. The next day, after removing the primary antibody on a magnet stand, the secondary antibody (Goat Anti‐Rabbit IgG, Vazyme, Ab207‐01) was diluted (1:100) in Dig‐wash Buffer and incubated with cells at room temperature for 1 h. Tagmentation was conducted by incubating cells with the pA/G‐Tnp Pro at room temperature for 1 h, followed by DNA fragmentation using TTBL at 37°C in a PCR thermal cycler. Spike‐in DNA was added as a control. The DNA product was isolated and purified using DNA Extract Beads Pro, terminated with a Stop Buffer at 95°C for 5 min and finally quantitatively assayed by qPCR. Sequences of primers used were listed in Table S3.

### 2.18 Statistical Analyses

ImageJ software was used to quantify the fluorescence intensity, grey value and predentin thickness. The GraphPad Prism 10.1.2 software was used for statistical analyses and visualisation. Data are presented as mean ± SEM. Comparisons between two groups used Student's *t*‐test, while among multiple groups applied one‐way ANOVA. *p* < 0.05 was considered statistically significant.

## 3 Results

### 3.1 Chromatin Remodeller BRD9 Interacts With RUNX2 and KLF4 in Nuclei of mDPCs

**FIGURE 1:**
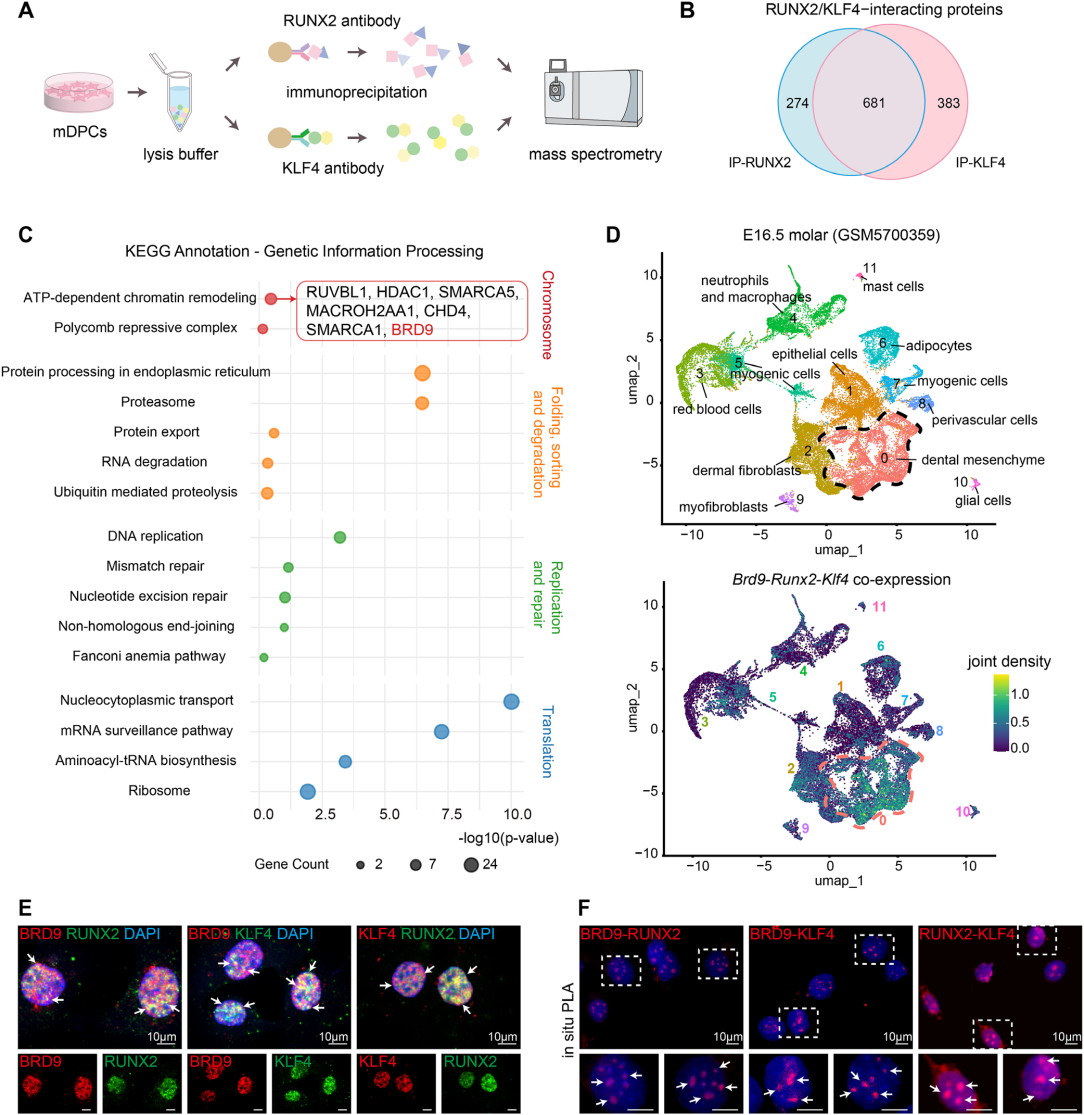
Identification of BRD9 and its interaction with RUNX2/KLF4. (A) Schematic workflow of the Co‐IP/MS strategy for identifying RUNX2 and KLF4 interacting proteins. (B) Venn diagram portrayed an overlap of proteins co‐immunoprecipitating with RUNX2 and KLF4. (C) KEGG annotation for common interactors of RUNX2 and KLF4 showed enrichment in chromatin remodelling. (D) Re‐analysis of published scRNA‐seq data for expression profiles in E16.5 mouse molar tissue showed co‐expression of *Brd9*, *Runx2* and *Klf4* in the dental mesenchyme. (E) Pairwise immunofluorescence images showed nuclear co‐localisation (white arrows) between BRD9, RUNX2 and KLF4 in E16.5 mDPC6T cells, as indicated by the yellow areas in merged images. From left to right: BRD9 (red)/RUNX2 (green); BRD9 (red)/KLF4 (green); KLF4 (red)/RUNX2 (green). (F) In situ PLA assays confirmed the direct interactions between BRD9, RUNX2 and KLF4. Red signals (white arrows) represented positive interactions. Nuclei were stained with DAPI (blue).

To identify the cofactors of RUNX2 and KLF4 in regulating odontoblastic differentiation, we performed Co‐IP using anti‐RUNX2 and anti‐KLF4 antibodies respectively in mDPC6T cells without odontoblastic differentiation induction, followed by mass spectrometry (MS) (Figure 1A). This analysis identified 955 RUNX2‐ and 1064 KLF4‐interacting proteins with high confidence, with a substantial overlap of 681 common proteins (Figure 1B; the list is provided in Table S4). KEGG analysis linked these shared interactors to ‘translation’, ‘folding, sorting and degradation’, ‘replication and repair’ and ‘chromosome’ within the primary category ‘Genetic Information Processing’ (Figure 1C).

Given the crucial role of chromatin remodelling in transcription program establishment, sustainment and termination [36], we prioritised the potential proteins within this term and focused on BRD9 as the primary candidate. Based on the re‐analysis of public single‐cell RNA‐seq datasets (GSM5700359) [37] for transcriptional landscapes in mouse molar tissue at E16.5, we detected the expression levels of above common RUNX2‐ and KLF4‐interacting proteins (Table S4). Most potential interactors displayed abundant expression within the dental mesenchymal cluster. Notably, *Brd9* was broadly detected across various clusters, while *Runx2* showed restricted yet high‐level expression specifically in the dental mesenchyme. *Klf4* exhibited its most prominent expression in fibroblasts, followed by a substantial presence in the dental mesenchyme (Figure S1A–D). Significantly, we observed their robust co‐expression within the dental mesenchyme (Figure 1D). Then, immunofluorescence staining assessed their subcellular localisation, with clear mutual colocalisation in the nuclei of mDPCs (Figure 1E). In situ PLA further visualised their interactions within close physical proximity, as evidenced by red fluorescent signals (Figure 1F). These results confirmed the BRD9‐RUNX2‐KLF4 interaction in the nuclei of mDPCs, suggesting their synergistic role in transcription regulation.

### 3.2 BRD9, RUNX2 and KLF4 Exhibit Divergent Spatiotemporal Expression Patterns During Odontoblastic Differentiation

**FIGURE 2:**
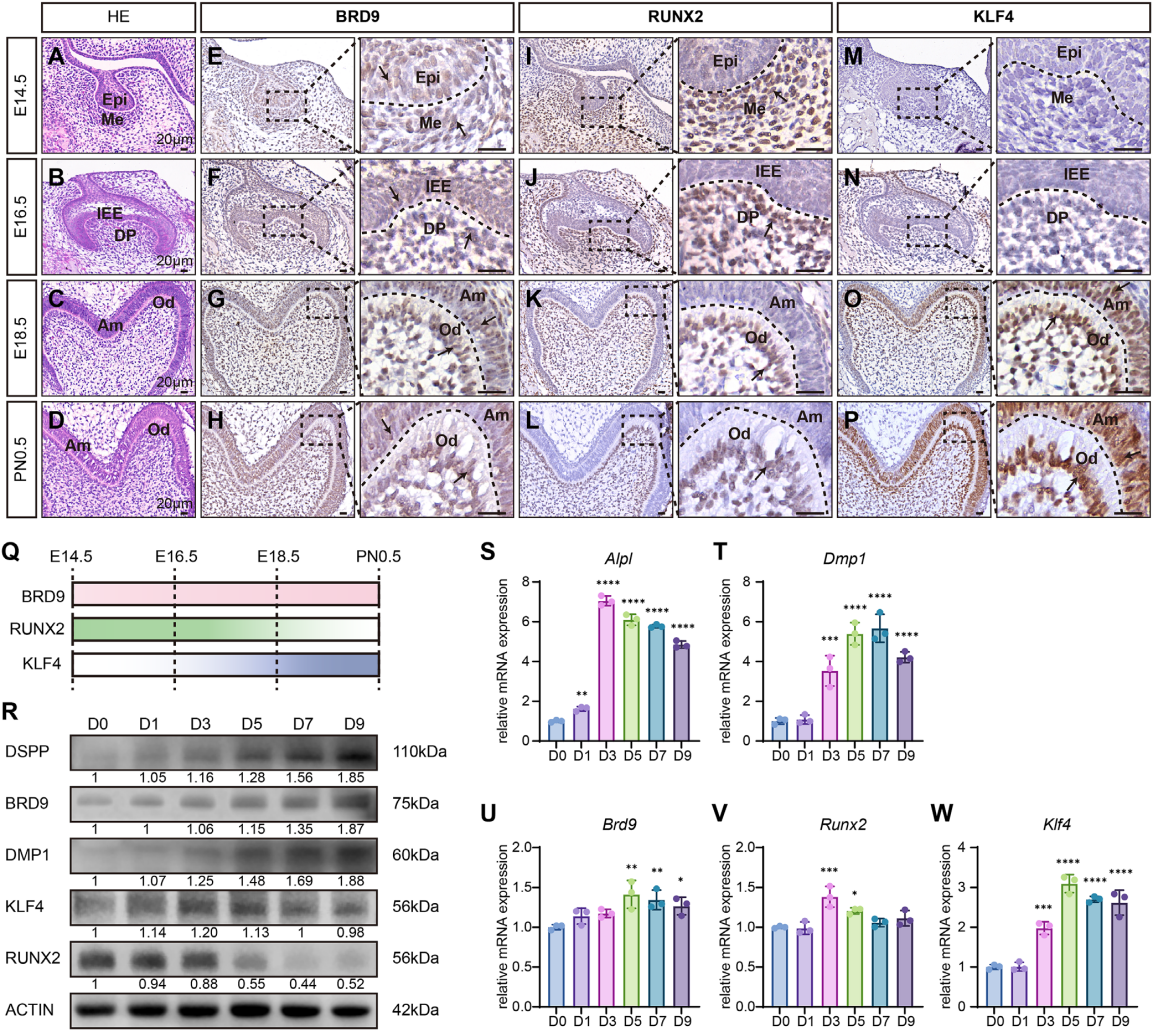
Expression profiling of BRD9, RUNX2 and KLF4 during odontoblastic differentiation in vivo and in vitro. (A–D) H&E staining portrayed the tooth morphology. (E–P) Immunohistochemistry (IHC) showed the expression patterns of BRD9, RUNX2 and KLF4 in mouse mandibular first molars at indicated developmental stages (E14.5, E16.5, E18.5 and PN0.5). Epi, epithelium; Me, mesenchyme; IEE, inner enamel epithelium; DP, dental papilla; Od, odontoblasts; Am, ameloblasts. Representative immunopositive cells (brown DAB signals) were indicated by arrows. (Q) Diagrammatic view illustrated the temporal dynamics of BRD9, RUNX2 and KLF4. (R) Western blot was performed to determine the protein levels of BRD9, RUNX2, KLF4 and odontoblastic markers (DSPP and DMP1) during odontoblastic differentiation induction for 9 days in vitro. (S–W) The mRNA expression levels of *Brd9*, *Runx2*, *Klf4*, *Dmp1*, *Dspp* and *Alpl* were examined by RT‐qPCR. *p < 0.05. **p < 0.01. ***p < 0.001. ****p < 0.0001.

We next explored the spatiotemporal expression pattern of BRD9, RUNX2 and KLF4 during tooth development via immunohistochemistry on mouse mandibular first molars at key stages (Figure 2A–P). BRD9 exhibited a widespread and constant nuclear localisation across all stages in both dental epithelial and mesenchymal lineages, including ameloblasts and odontoblasts (Figure 2E–H). In contrast, the transcription factors showed divergent and stage‐specific expression. RUNX2 was intensively expressed in dental mesenchyme and papilla during early cap (E14.5) and bell (E16.5) stages, but gradually declined in differentiated odontoblasts from late bell stage (E18.5) onwards (Figure 2I–L). Conversely, KLF4 was weakly expressed initially (at E14.5, E16.5) but markedly upregulated in terminally differentiated odontoblasts and ameloblasts at E18.5 and PN0.5 (Figure 2M–P). Their temporal shifts were intuitively portrayed using a colour gradient in the diagrammatic view (Figure 2Q).

To further evaluate their expression dynamics in vitro, E16.5 mDPCs were cultured under odontoblastic induction for 9 days. The induction successfully triggered odontoblastic differentiation, verified by upregulation of differentiation markers (Figure 2R–T). Mirroring the in vivo data, *Brd9* increased at both protein and mRNA levels (Figure 2R,U). Consistently, *Runx2* was prominently expressed in early 3 days, then declined but remained detectable (Figure 2R,V), while *Klf4* upregulated progressively as the differentiation progressed, peaking at day 5 (Figure 2R,W).

Collectively, these results reveal a dynamic shift in the expression of the transcription complex: BRD9 remains constantly expressed, while RUNX2 peaks early and KLF4 peaks late (Figure 2Q). The persistence of RUNX2 expression at later stages, concomitant with significant upregulation of KLF4 at terminal stages, suggests their potential cooperation during odontoblastic differentiation.

### 3.3 Neural Crest‐Specific Ablation of *Brd9* or *Runx2*/*Klf4* Leads to Disordered Odontoblastic Differentiation In Vivo

**FIGURE 3:**
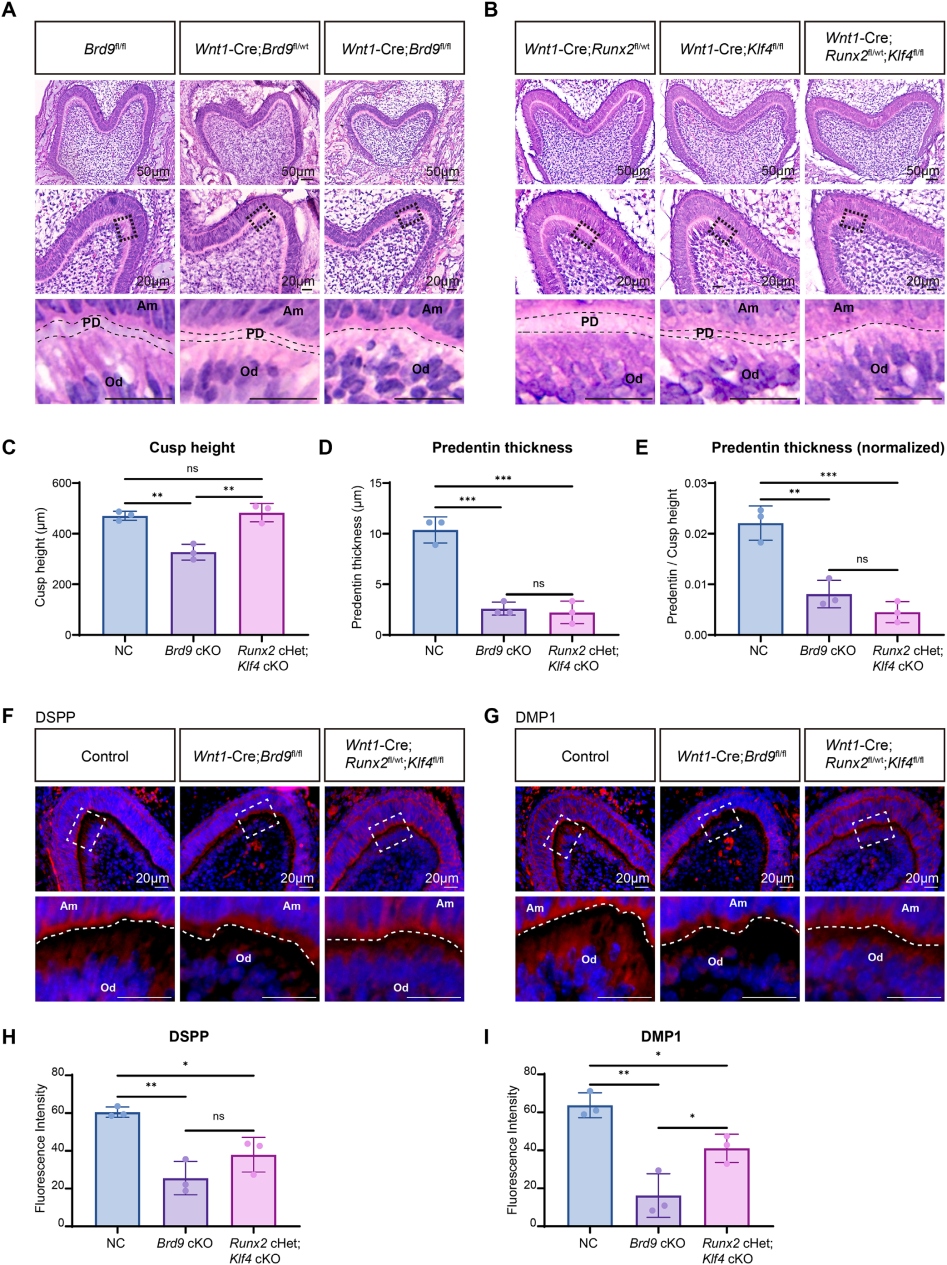
Neural crest‐specific ablation of *Brd9* or *Runx2/Klf4* results in disordered odontoblastic differentiation. (A, B) H&E staining for tooth morphology of *Wnt1*‐Cre; *Brd9* ^fl/fl^ and *Wnt1*‐Cre; *Runx2* ^fl/wt^; *Klf4* ^fl/fl^ mice at PN0.5. They both exhibited disordered odontoblast layers with scant predentin formation. Od, odontoblasts; Am, ameloblasts; PD, predentin. (C) Quantification of cusp height. *Wnt1*‐Cre; *Brd9* ^fl/fl^ mice showed reduced cusp heights, while *Wnt1*‐Cre; *Runx2* ^fl/wt^; *Klf4* ^fl/fl^ mice exhibited no significant alteration compared to controls. (D, E) Quantification of absolute predentin thickness (D) and predentin thickness normalised to cusp height (E) at PN0.5. Both parameters were significantly reduced in mutant mice, indicating impaired dentinogenesis. (F, G) Immunofluorescence showed decreased expression of DMP1 and DSPP protein in the odontoblast layer of molar cusps of mutant mice. (H, I) Quantitative analysis of DSPP and DMP1 protein fluorescence intensity. *p < 0.05. **p < 0.01. ****p < 0.0001. ns (*p* > 0.05), not significant.

To elucidate the function of *Brd9* in odontogenesis, we generated neural crest‐specific knockout mice using *Wnt1*‐Cre, which marked the dental papilla cells and odontoblasts derived from migrating CNC cells [38]. *Wnt1*‐Cre; *Brd9* ^fl/fl^ (*Brd9* cKO) mutants died neonatally with cleft palates (Figure S2A–J). IHC confirmed efficient BRD9 ablation in the dental mesenchyme (Figure S3A,B). Their molars displayed reduced sizes characterised by reduced cusp height, disorganised odontoblast polarity with markedly reduced predentin formation, while *Wnt1*‐Cre; *Brd9* ^fl/wt^ (*Brd9* cHet) showed milder disarray (Figure 3A). Early stages (E15, E17) revealed no obvious abnormality in unpolarised papilla cells (Figure S4A,B). To quantitatively assess the subtle morphological defects, we measured the cusp height and epithelial invagination depth at embryonic days. The *Brd9* cKO molar germs exhibited a reduction in both parameters at E17, the bell stage when odontoblastic differentiation initiated, suggesting its function in early tooth morphogenesis (Figure S4E,F).

To distinguish whether the observed defects stem from early progenitor pool attrition, we detected the expression of key markers at pre‐differentiation stages (Figure S5A,C). The percentage of positive area and cell density for SIX2, PAX9 and Ki67 in condensed mesenchyme at E12.5, as well as for PAX9, MSX1, Ki67 and Cleaved Caspase‐3 (CC‐3) in the dental papilla at E15, were comparable between control and *Brd9* cKO embryos (Figure S5B,D). These results indicate that the progenitor identity, proliferation and survival of CNC‐derived dental mesenchymal cells are preserved upon *Brd9* ablation, suggesting that BRD9 is specifically required for the terminal odontoblast differentiation program.

Since *Klf4* single knockout impairs mineralisation but not the odontoblast morphology [13], while *Runx2* ablation arrests the tooth germ development early at the bud stage [39, 40], we generated compound *Wnt1*‐Cre; *Runx2* ^fl/wt^; *Klf4* ^fl/fl^ (*Runx2* cHet; *Klf4* cKO) mice to detect their synergistic relationship in vivo. These mutants exhibited a marked reduction in RUNX2 expression with concomitant ablation of KLF4 (Figure S3C–F). Interestingly, single mutants retained polarised odontoblast morphology and defined predentin layer formation at PN0.5, while the compound mutants exhibited a mild disorder in odontoblast polarisation with scant predentin formation, despite maintaining a virtually normal cusp height (Figure 3B,C), indicating that heterozygous *Runx2* mutation exacerbated odontoblastic defects caused by *Klf4* ablation, particularly in the formation of mineralised dentine tissue.

Immunofluorescence further showed downregulation of DSPP and DMP1 in the odontoblast cytoplasm of *Runx2* cHet; *Klf4* cKO and an even more pronounced reduction in *Brd9* cKO (Figure 3F–I). Consistently, though DSPP and DMP1 were almost undetectable at E15, they were downregulated within the dental papilla at E17 upon *Brd9* ablation (Figure S4C,D,G). Statistical analysis of predentin thickness further revealed that dentinogenesis was suppressed in both mutants (Figure 3D,E). These results confirm that *Brd9* or *Runx2*/*Klf4* ablation attenuates the differentiation and maturation of odontoblasts, thereby inhibiting the secretion and mineralisation of dentine matrix.

Moreover, we observed no significant difference in RUNX2 or KLF4 protein levels between the *Brd9* cKO and control samples (Figure S6A–D), suggesting that BRD9 did not directly regulate the expression of RUNX2/KLF4 but shared a similar biological function with RUNX2‐KLF4 complex in the process of odontoblastic differentiation and dentinogenesis.

Taken together, RUNX2 and KLF4 act synergistically to promote odontoblast maturation and predentin formation, while the fact that *Brd9* loss results in a similar and even more severe phenotype strongly supports BRD9 as a critical upstream coordinator of RUNX2‐KLF4 activity during odontoblast lineage commitment.

### 3.4 Chemical BRD9 Degradation Inhibits Odontoblastic Differentiation In Vitro

**FIGURE 4:**
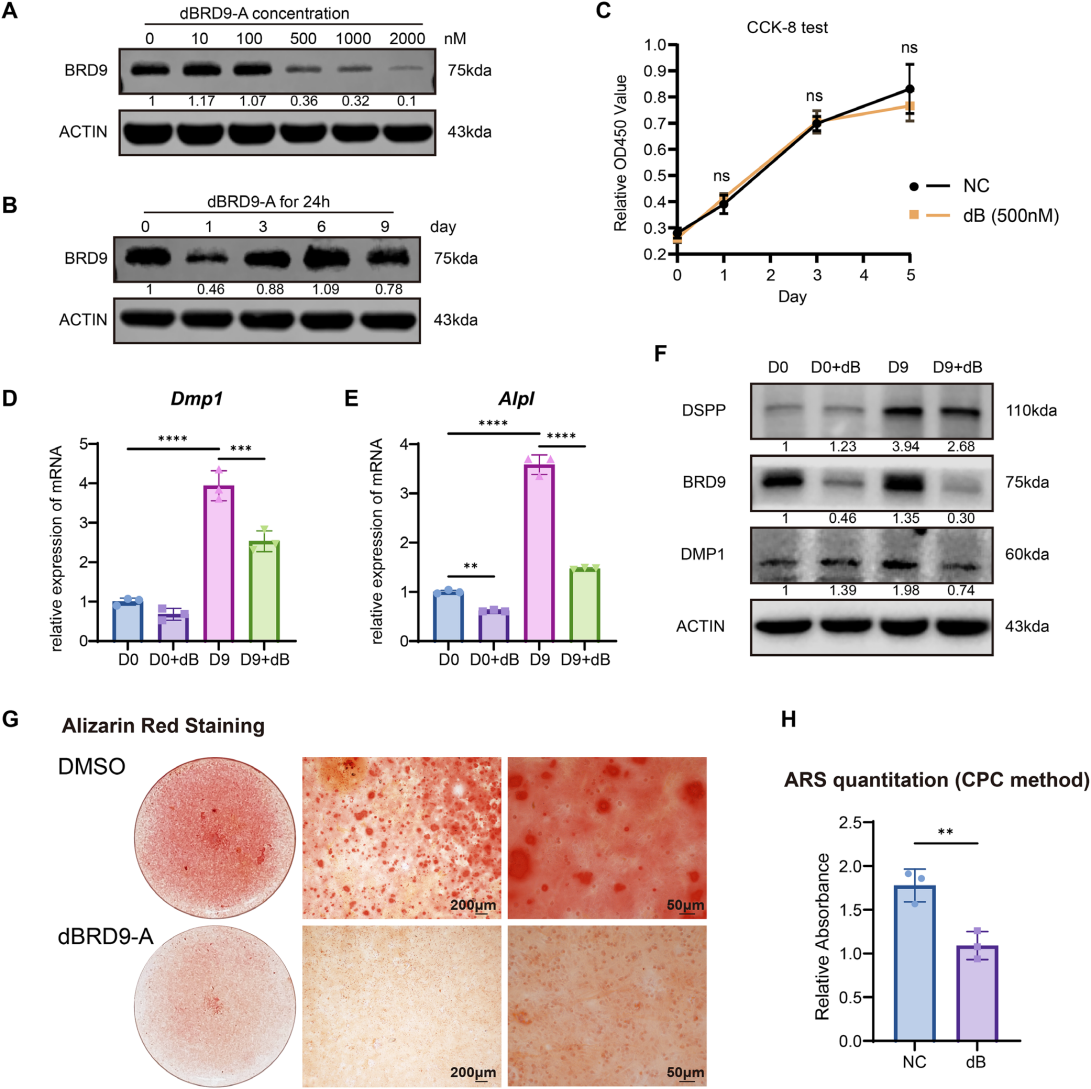
Chemical BRD9 degradation inhibits the odontoblastic differentiation and mineralised nodule formation in vitro. (A) Western blot for BRD9 expression at different dBRD9‐A concentrations showed that the chemical degradation achieved in a dose‐dependent manner. (B) Western blot for BRD9 expression after 24 h‐treatment by dBRD9‐A revealed that it recovered after dBRD9‐A withdrawal. (C) CCK‐8 test showed no significant difference in cell proliferation upon BRD9 continuous degradation. (D, E) The qRT‐PCR results showed downregulated mRNA levels of *Dmp1* and *Alpl* in dBRD9‐A group at D9. (F) Western blot analysis revealed downregulated expression of DSPP and DMP1 upon BRD9 degradation after odontoblastic differentiation induction. (G) Fewer mineralised nodules were observed in dBRD9‐A treated mDPCs by alizarin red staining. (H) Quantitative analysis of alizarin red staining using CPC method. **p < 0.01. ***p < 0.001. ****p < 0.0001. ns (*p* > 0.05), not significant.

To investigate the potential of BRD9 in odontoblastic differentiation in vitro, we treated mDPCs with a chemical degrader dBRD9‐A to acutely deplete BRD9 protein levels. This molecule is an optimised BRD9‐specific PROTAC that exhibits potent and selective BRD9 degradation properties across a broad concentration range [23, 24, 41]. After testing the protein degradation efficiency under a concentration gradient (0, 10, 100, 500, 1000, 2000 nM) for 24 h, we selected 500 nM for optimal degradation (Figure 4A) and we observed BRD9 expression recovered after dBRD9‐A withdrawal (Figure 4B), indicating that sustained exposure was necessary for stable degradation. Besides, this effective dBRD9‐A administration had no detectable effect on cell proliferation (Figure 4C). Under sustained treatment with 500 nM dBRD9‐A, odontoblastic differentiation over 9 days was markedly impaired compared to the control, with a significant suppression of differentiation markers (DMP1, DSPP and ALPL) (Figure 4D–F). Furthermore, alizarin red staining and subsequent quantification demonstrated a substantial reduction of calcified nodule formation upon BRD9 loss (Figure 4G,H). Despite the marked differentiation defects, BRD9 degradation did not significantly alter RUNX2 and KLF4 expression at D5 (Figure S7A,B). These findings suggest that BRD9 is cell‐intrinsically essential for activating odontoblastic differentiation and mineralisation capacity, corroborating the dentinogenic defects observed in mice.

### 3.5 Loss of BRD9 Downregulates Transcripts and Chromatin Accessibility Associated With Odontogenesis and Mineralisation

**FIGURE 5:**
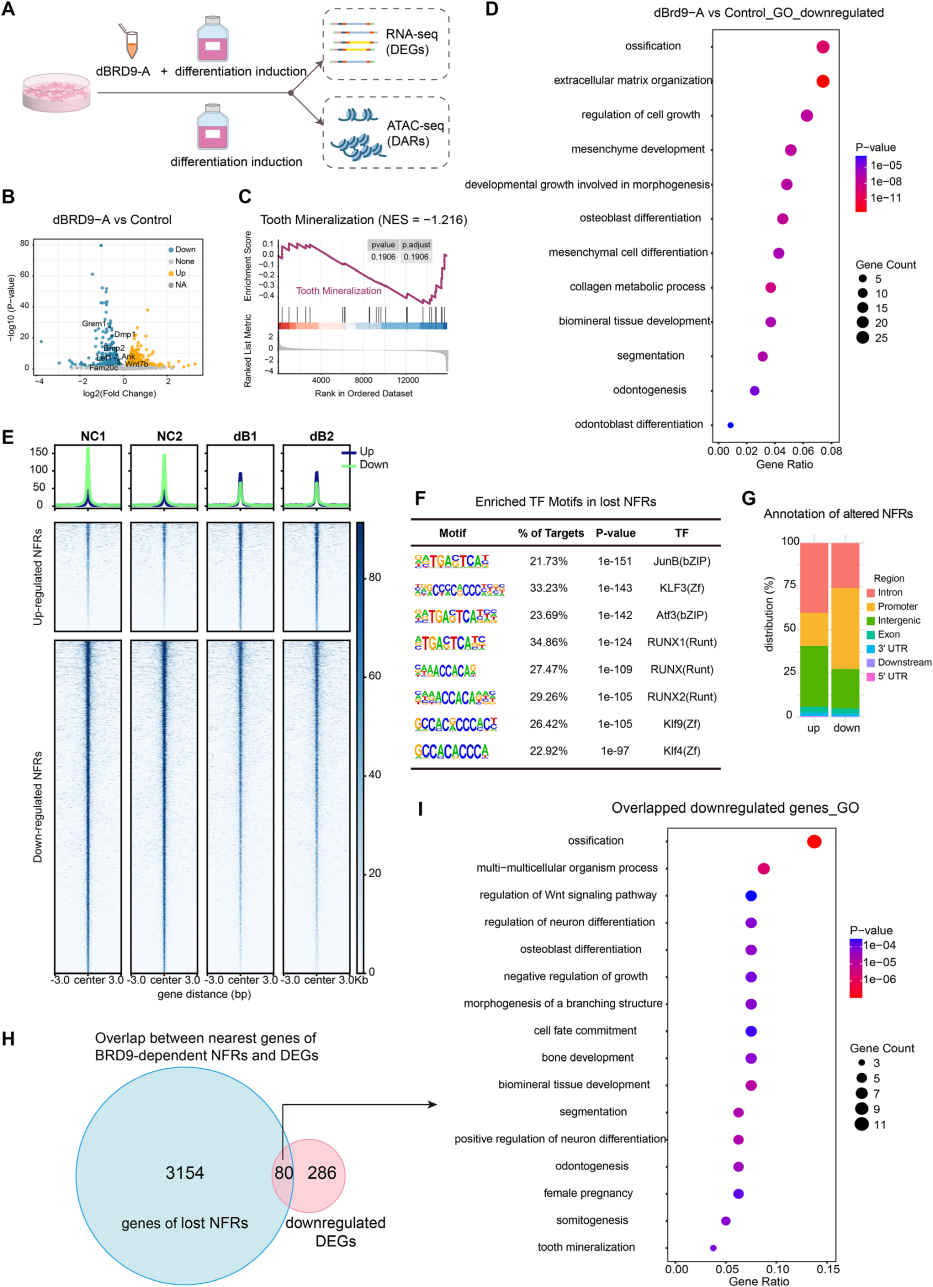
Genome wide profile of BRD9‐dependent genes and genomic accessibility are associated with odontogenesis and mineralisation during odontoblastic differentiation. (A) Schematic representation of experimental workflow. (B) Scatter plot showed the genes up‐ or downregulated by dBRD9‐A in odontoblastic induced mDPCs. *p*‐values and fold changes for each gene were generated by sleuth. (C) Gene set enrichment analysis (GSEA) revealed an overall downregulated trend (NES = −1.216) of gene sets related to tooth mineralisation upon BRD9 degradation. NES, Normalised Enrichment Score. (D) Dot plot showed the Gene Ontology (GO) enrichment assay for downregulated genes from RNA‐seq profiles. (E) Nucleosome‐free region (NFR) summit‐centred heatmap of ATAC‐seq signals in the control and dBRD9‐A group (NC, *n* = 2; dB, *n* = 3). Data from two representative replicates per group are shown for clarity. (F) Sheet for the top enriched transcription factor motifs in BRD9‐dependent NFRs. (G) Genomic distribution of up‐ and downregulated NFRs. (H) Integration analyses of RNA‐seq and ATAC‐seq showed 80 genes with concurrent downregulation of expression and reduction in chromatin accessibility. (I) GO enrichment for the integrated targeted genes upon BRD9 degradation.

To delineate BRD9‐dependent regulatory mechanisms during odontoblastic differentiation, here we employed a multi‐omics strategy integrating transcriptomic and epigenomic profiles. E16.5 mDPCs were cultured under odontoblastic differentiation induction for 5 days with or without dBRD9‐A treatment and were harvested for RNA‐seq and ATAC‐seq respectively (Figure 5A).

RNA‐seq identified 793 differentially expressed genes, including 427 upregulated and 366 downregulated genes upon BRD9 degradation (Figure 5B). Notably, *Runx2* and *Klf4* transcript levels were not significantly altered upon BRD9 degradation (Figure S8A). The detected upregulated transcripts were enriched in cell cycle, chromosome segregation and DNA replication by GO and KEGG pathway analyses (Figure S8B,C), while significantly downregulated genes were enriched in categories associated with mesenchyme development and mineralisation, such as ossification, extracellular matrix organisation, osteoblast differentiation and odontogenesis (Figure 5D) and in signalling pathways involving TGF‐β and Wnt (Figure S8D). Notably, for global transcriptional changes, GSEA confirmed the suppression of gene sets involved in tooth mineralisation and extracellular matrix organisation (NES < 0) (Figure 5C, Figure S8E). Heatmap analysis revealed that odontogenesis‐related genes (including *Dmp1*, *Bmp2*, *Lef1*, *Wnt7b*, *Sp6* and *Fam20c*) were downregulated upon BRD9 loss (Figure S8F). These results established BRD9 as a positive transcriptional regulator for odontogenesis and mineralisation.

With ATAC‐seq, we identified 3809 lost NFRs (nucleosome‐free regions) and 1246 gained NFRs in dBRD9‐A group compared with the control (analysis based on *n* = 2 for NC and *n* = 3 for dB) (Figure 5E), underscoring that BRD9 is predominantly responsible for opening the folded nucleosomes and increasing genomic chromatin accessibility. HOMER analysis was performed to discover the highly enriched TF motifs in BRD9‐dependent accessible regions. Except for the bZIP family that was close with odontogenesis, TFs from the RUNX and KLF family including RUNX2 and KLF4 were ranked at the top (Figure 5F), providing mechanistic insight into BRD9's coordination with RUNX2 and KLF4. Then, lost NFRs were annotated by HOMER to predict their target genes and assign their genomic locations. The distribution diagram showed that these BRD9‐dependent NFRs were primarily located in promoter and distal intergenic regions (Figure 5G). Integration of both datasets identified 80 genes with coupled downregulation in chromatin accessibility and transcript levels upon BRD9 degradation (Figure 5H) and they were functionally linked to ossification, osteoblast differentiation, cell fate commitment, biomineral tissue development and odontogenesis (Figure 5I). Key genes within odontogenesis included *Ank*, *Lef1*, *Dmp1*, *Fam20c* and *Apcdd1*.

Taken together, above results establish BRD9 as a critical epigenetic regulator to orchestrate odontogenesis by maintaining the chromatin accessibility at key regulatory elements, potentially through its functional interplay with RUNX2 and KLF4 in transcriptional activation.

### 3.6 BRD9 Maintains *Fam20c* Expression by Facilitating the Binding of RUNX2 and KLF4 to Its Enhancer

**FIGURE 6:**
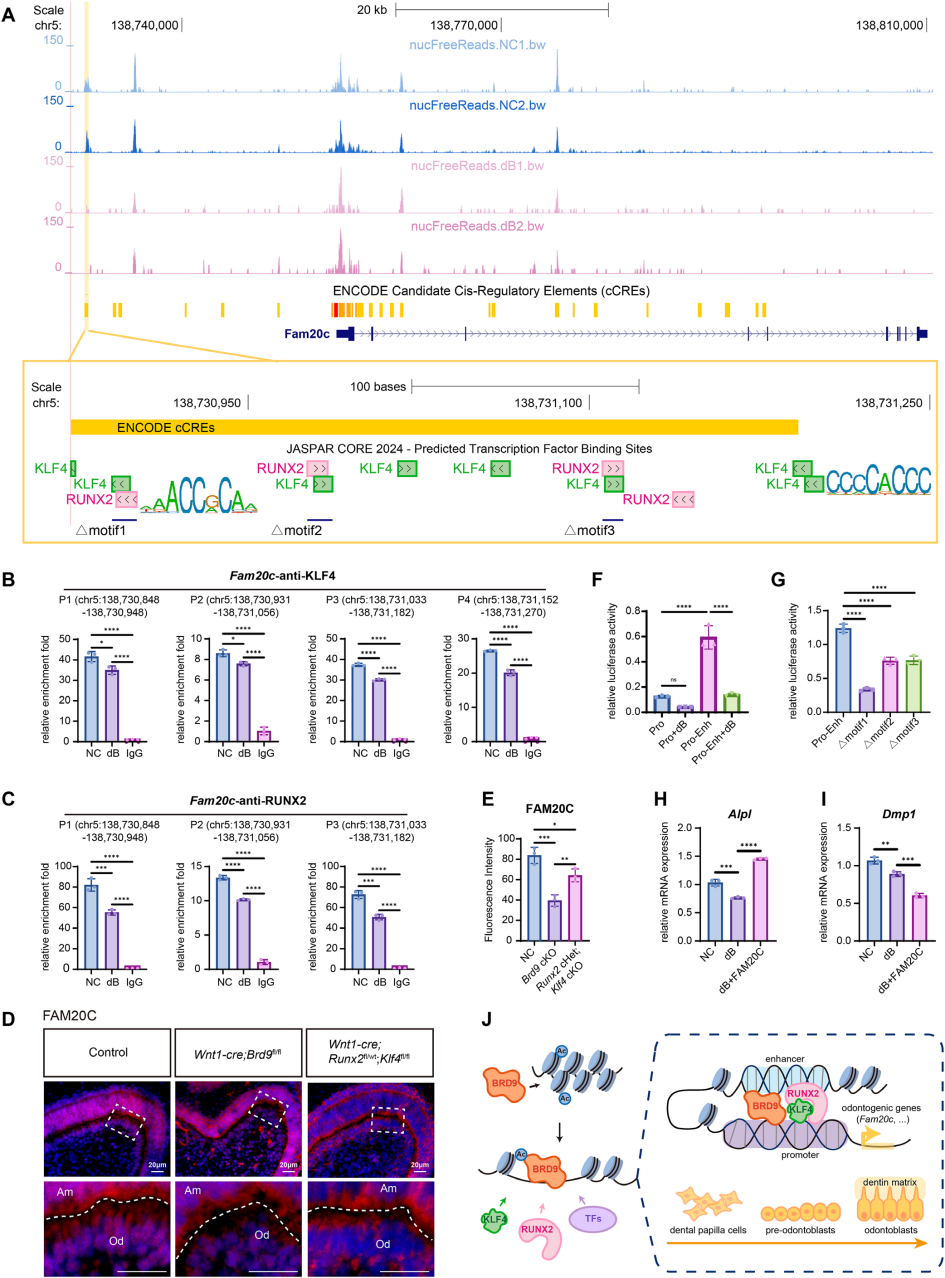
BRD9 coordinates RUNX2 and KLF4 at *Fam20c* enhancer loci for transcription activation. (A) Visualisation of ATAC‐seq results at BRD9‐dependent intergenic regions of *Fam20c*. Data from two representative replicates per group were displayed. (B, C) CUT&Tag‐qPCR results showed decreased RUNX2 and KLF4 occupancy at *Fam20c*‐related NFR upon BRD9 degradation. (D) Mice with *Brd9* or *Runx2*/*Klf4* ablation exhibited reduced FAM20C expression within the odontoblast layer. (E) Quantitative analysis of FAM20C protein fluorescence intensity. (F) Dual‐luciferase reporter assay revealed the enhancer activity of the *Fam20c*‐related NFR. (G) Deletion of RUNX2/KLF4 motifs within the *Fam20c* enhancer downregulated its transcription activity. (H, I) The qRT‐PCR results showed that adding recombinant mFAM20c protein recovered the expression of *Alpl* but not *Dmp1* upon dBRD9‐A treatment, suggesting a partial rescue of odontoblastic differentiation capacity. (J) Schematic model of BRD9‐mediated dynamic transcription factor recruitment for odontoblastic differentiation. BRD9‐mediated chromatin remodelling establishes accessible chromatin landscapes and facilitates the binding of RUNX2‐KLF4 to the *Fam20c* enhancer, enabling cooperative transcriptional activation. *p < 0.05. **p < 0.01. ***p < 0.001. ****p < 0.0001.

The integrated analyses supported that BRD9 was responsible for the expression and accessibility of several key odontogenic genes (e.g., *Dmp1*, *Lef1*, *Fam20c*). Among these, *Lef1* is a well‐established mediator of WNT and FGF signalling critical for tooth morphogenesis [42, 43]. *Fam20c*, also termed *Dmp4*, encodes a secreted kinase essential for differentiation and dentine matrix formation [44]. Inactivation of *Fam20c* leads to remarkable dentine defects with DSPP, DMP1 loss [45, 46]. Therefore, to determine how BRD9 coordinates RUNX2‐KLF4 at specific loci, we analysed BRD9‐sensitive NFRs annotated to these genes (Figure 6A, Figure S9A,B) and conducted CUT&Tag‐qPCR for regions enriched with predicted RUNX2 and KLF4 motifs. BRD9 degradation reduced RUNX2 binding at the intergenic loci of *Dmp1* and *Lef1* without affecting KLF4 occupancy (Figure S9C,D), while in *Fam20c*‐related region, both RUNX2 and KLF4 binding were markedly diminished (Figure 6B,C), indicating that BRD9‐mediated chromatin opening facilitated RUNX2‐KLF4 co‐recruitment. Furthermore, through the dual‐luciferase assay, this intergenic region exhibited an enhancer activity, which was significantly inhibited upon BRD9 loss (Figure 6F). Deletions of RUNX2‐KLF4 binding motifs within this region distinctly downregulated its transcriptional activity (Figure 6G).

Consistent with above findings, molars of *Brd9* cKO and *Runx2* cHet; *Klf4* cKO mice displayed reduced FAM20C signals within odontoblast layers (Figure 6D,E). In vitro supplementation with exogenous recombinant FAM20C protein incompletely rescued the differentiation blockade caused by BRD9 degradation, as demonstrated by a significant transcriptional recovery of *Alpl* (Figure 6H), though *Dmp1* remained suppressed (Figure 6I), suggesting that FAM20C may be a crucial but not exclusive downstream effector for BRD9.

Collectively, BRD9 acts as an upstream epigenetic gatekeeper that establishes accessible chromatin landscapes for transcription factor recruitment. Specifically, it facilitates the dynamic binding of RUNX2 and KLF4 to the *Fam20c* enhancer for cooperative transcriptional activation, thereby safeguarding the odontoblast lineage commitment and dentinogenesis (Figure 6J).

## 4 Discussion

In eukaryotes, lineage commitment is orchestrated by chromatin regulators and ambient transcription factors cooperating within complex and precise networks. Functional perturbations of chromatin remodellers give rise to various developmental defects ranging from embryonic development to organogenesis [47]. While RUNX2 and KLF4 are established critical TFs guiding odontoblastic differentiation, chromatin dynamics underlying their transcriptional specificity remain indistinct. Our study identifies BRD9 as an essential epigenetic coordinator. In other biological contexts such as haematopoiesis, osteoclastogenesis and pluripotency maintenance, BRD9 alters chromatin accessibility to regulate the occupancy and activity of lineage‐determining TFs like CTCF [48], GATA1 [49], FOXP1 [50], KLF4, SP5 and SMAD2/3 [19, 20]. Reversely, BRD9 is recruited by SMAD2/3 to specific loci for enhancer‐promoter connectome [51]. These findings position BRD9 as a pivotal hub linking the chromatin state to transcriptional output. Our work defines the functional crosstalk between BRD9 and RUNX2‐KLF4 complex, thereby establishing a novel transcriptional regulatory model for odontoblast lineage commitment. During odontoblastic differentiation, *Runx2* expression peaks at the early stage and then declines. This is consistent with its spatiotemporal regulation in *Dspp* promoter activity [12], suggesting the critical role of RUNX2 in odontoblast lineage commitment. In contrast, *Klf4* expression progressively increases throughout differentiation, aligning with its function in dentinogenesis [13]. The distinct expression dynamics of RUNX2 and KLF4 indicates their sequential cooperation for differentiation. Here, we ulteriorly demonstrated a critical synergy between RUNX2 and KLF4 in vivo, as compound mutants (*Wnt1*‐Cre; *Runx2* ^fl/wt^; *Klf4* ^fl/fl^ mice) exhibited odontoblast and predentin disorganisation not observed in single knockouts. Notably, this defect was milder than that caused by *Brd9* ablation. This phenotypic hierarchy indicates that though BRD9 is essential for coordinating RUNX2‐KLF4 complex to activate odontoblastic differentiation, it prefers to perform more overall functions beyond RUNX2‐KLF4 axis. We speculate that it may maintain a global permissive chromatin landscape or coordinate additional TF networks.

To further dissect the functional output of BRD9‐RUNX2/KLF4, we performed rescue experiments with recombinant FAM20C. The divergent rescue responses—*Alpl* recovery but persistent *Dmp1* suppression highlight the complexity of regulatory networks. FAM20C‐dependent compensation is sufficient to restore partial differentiation markers, but is inadequate to fully rescue the odontoblast maturation and matrix mineralisation program. These suggest a hierarchical model wherein BRD9, on the one hand, promotes differentiation via activating *Fam20c* with RUNX2‐KLF4 and on the other hand, functions through FAM20C‐independent pathways. The latter is consistent with its role in maintaining global chromatin accessibility and facilitating transcription factor occupancy at odontogenic gene loci.

Furthermore, previous studies have reported that SWI/SNF complexes target distal enhancers and cooperate with p300 to modulate H3K27ac [52]. In osteoclastogenesis, ARID1A‐ and BRD9‐containing complexes play a functional antagonism in transcription regulation, both of which depend on BRD4 coordination [53]. Whether BRD9 similarly recruits specific histone modifiers at specific loci and how it interfaces with other chromatin remodellers to fine‐tune odontogenesis and other processes are compelling questions for future in‐depth study. The pleiotropic functions of BRD9, together with the compensatory and feedback mechanisms across abundant pathways pose challenges for therapeutic targeting, yet also opportunities for insights into the underlying intricate regulatory networks. Given that *Brd9* was knockout across multiple lineages using *Wnt1*‐Cre, the observed cleft palate suggests a broader necessity of BRD9 in CNC‐derived mesenchyme development beyond odontoblastic differentiation. To circumvent the blockade of neonatal lethality, future studies using dental mesenchyme‐specific knock‐out like *Dmp1*‐Cre, or taking the strategy of transplanting tooth germ under kidney capsule, will allow subsequent investigation of postnatal dentinogenesis and later‐stage tooth development. This limitation in mice also indicates that BRD9 plays a more extensive role in oral and craniofacial development.

Although no direct pathogenic variants in the *BRD9* gene have been reported to date, its critical role in skeletal development supports its potential in related craniofacial deformity [21]. Mutations in other SWI/SNF complex subunits are well‐established causes of a spectrum of human developmental disorders collectively termed ‘BAFopathies’ [54, 55]. Variants in another ncBAF‐specific component, *GLTSCR1*, have been identified as the pathogeny for a series of rare neurodevelopmental disorders with coarse facial features [56]. Notably, cytogenetic location of *BRD9* at 5p15.33 is mapped to the critical region of the Cri du chat syndrome (CdCS), a genetic disease with typical craniofacial and oral features [57, 58]. And here our findings further raise the possibility that BRD9 dysfunction may contribute to human dental and palatal phenotypes. By defining the BRD9‐RUNX2‐KLF4 coordination, our work provides a concrete mechanistic model for understanding the interplay between lineage‐specific transcription factors and chromatin remodellers in development and disease, warranting future genetic studies in chromatin‐associated human dental and craniofacial developmental defects and the underlying intricate multi‐dimensional mechanisms.

## 5 Conclusion

Our study delineates a multi‐dimensional epigenetic‐transcriptional network wherein BRD9 acts as a core coordinator, shaping the chromatin landscape to facilitate RUNX2‐KLF4 binding, thereby activating downstream effectors such as *Fam20c* to drive odontoblastic differentiation and matrix protein secretion (Figure 6J). This accounts for the disordered odontoblast morphology and reduced predentin formation observed in mice with *Brd9* or *Runx2*/*Klf4* ablation. This discovery not only deepens our understanding of the molecular mechanisms in tooth development, but also holds clinical promise. Clarifying the role of the BRD9‐RUNX2‐KLF4 in dentinogenesis may provide innovative molecular targets for diagnosing dentine dysplasia and lay a theoretical foundation in future strategies for dentine regeneration based on epigenetic and transcriptional interventions.

## Author Contributions

W.Z. and Y.L. contributed to experiment design, data acquisition, analysis, interpretation, drafted and revised the manuscript. Y.G., Y.L. and H.R. contributed to data acquisition and analysis. D.H. contributed to data analysis and interpretation. G.Y. contributed to data interpretation and critically revised the manuscript; H.L. and Z.C. contributed to conception, design, data interpretation, critically revised the manuscript. All authors gave final approval and agreed to be accountable for all aspects of the study.

## Funding

This study was supported by the National Natural Science Foundation of China (No. 82230029 and 82470938) to Zhi Chen; the National Natural Science Foundation of China (No. 82322014 and 82270948) and the Interdisciplinary Research Project of School of Stomatology Wuhan University (No. XNJC202306) to Huan Liu; the National Natural Science Foundation of China (No. 82501091) to Yuxiu Lin.

## Ethics Statement

Animal studies were approved by the Institutional Animal Care and Use Committees at School and Hospital of Stomatology of Wuhan University (No. S07924120J).

## Conflicts of Interest

The authors declare no conflicts of interest.

## Supporting information


**Figure S1:** The transcriptomic profiling of scRNA‐seq in E16.5 mouse molar characterised the expression of *Brd9*, *Runx2*, *Klf4* and signature markers. (A–C) Feature plot of *Brd9*, *Runx2* and *Klf4* in different cell populations. (D) Dot plot of signature genes in the cell populations.
**Figure S2:** Neural crest‐specific ablation of *Brd9* results in cleft palate. (A–D) H&E staining for middle palate morphology at E15 and E17. *Brd9* cKO mice revealed a failure in palatal shelf fusion since E15. (E–J) H&E staining for palate morphology at PN0.5. *Brd9* cKO mice exhibited a complete cleft palate after birth. A, anterior palate; M, middle palate; P, posterior palate. Dashed lines referred to the midline where the palatal suture was located.
**Figure S3:** Expression pattern of BRD9, RUNX2 and KLF4 in mandibular first molars of *Wnt1*‐Cre; *Brd9* ^fl/fl^ and *Wnt1*‐Cre; *Runx2* ^fl/wt^; *Klf4* ^fl/fl^ mice. (A, B) *Wnt1*‐Cre; *Brd9* ^fl/fl^ mice revealed distinct ablation of BRD9 in the dental papilla. (C–F) *Wnt1*‐Cre; *Runx2* ^fl/wt^; *Klf4* ^fl/fl^ mice showed KLF4 ablation and RUNX2 downregulation in the dental mesenchyme. Od, odontoblasts; Am, ameloblasts. Representative immunopositive cells (brown DAB signals) are indicated by arrows.
**Figure S4:** Loss of *Brd9* influences early tooth development. (A, B) H&E staining for tooth germs of control and *Brd9* cKO mice at E15 and E17. DP, dental papilla. (C, D) Immunofluorescence of DSPP and DMP1 protein in molar germs. (E, F) Quantification of cusp height and normalised epithelial invagination depth in control and cKO tooth germs at E15 and E17. (G) Quantitative analysis of DMP1 and DSPP protein fluorescence intensity in dental papilla at E17. **p < 0.01. *** *p* < 0.001. ns (*p* > 0.05), not significant.
**Figure S5:** Loss of Brd9 does not affect early dental mesenchyme progenitor maintenance. (A) Immunofluorescence of SIX2, PAX9, Ki67 and CC‐3 (Cleaved Caspase‐3) proteins in the condensed dental mesenchyme at E12.5. DM, dental mesenchyme; DE, dental epithelium. (B) Quantitative analysis of the percentage of marker‐positive area and marker‐positive cell density (cells/100 μm^2^) in DM at E12.5. (C) Immunofluorescence of MSX1, PAX9, Ki67 and CC‐3 in the dental papilla at E15. DP, dental papilla; EO, enamel organ. (D) Quantitative analysis of percentage of marker‐positive area and marker‐positive cell density (cells/100 μm^2^) in DP at E15. No significant differences were detected between the control and *Brd9* cKO embryos for the expression of above markers. ns (*p* > 0.05), not significant.
**Figure S6:** Neural crest ablation of *Brd9* does not alter the expression of RUNX2 and KLF4 in the odontoblast layer. (A, B) Immunofluorescence of RUNX2 and KLF4 in molar cusps of control and *Brd9* cKO mice. (C, D) Quantitative analysis of DMP1 and DSPP protein fluorescence intensity. ns (*p* > 0.05), not significant.
**Figure S7:** BRD9 degradation does not affect RUNX2 or KLF4 expression during odontoblastic differentiation in vitro. (A) Western blot for RUNX2 and KLF4 expression at D5 of odontoblastic induction. (B) Quantitative analysis of RUNX2 and KLF4 protein levels. ns (*p* > 0.05), not significant.
**Figure S8:** Transcriptomic analysis for dBRD9‐A treated mDPCs at Day 5. (A) Transcript levels of *Runx2* and *Klf4* at D5. ns (*p* > 0.05), not significant. (B) GO enrichment for upregulated genes upon BRD9 degradation. (C) KEGG pathway enrichment for upregulated genes. (D) KEGG pathway enrichment analysis revealed that TGF‐β and Wnt signalling pathway were downregulated. (E) GSEA indicated significant downregulation of the extracellular matrix organisation pathway. NES, Normalised Enrichment Score. (F) Heatmap of odontogenesis‐related genes in NC and dB groups.
**Figure S9:** BRD9 maintains chromatin accessibility at odontogenic gene loci. (A, B) Visualisation of ATAC‐seq results at BRD9‐dependent intergenic regions of *Dmp1* and *Lef1*. Data from two representative replicates per group were displayed. (C, D) CUT&Tag‐qPCR results showed that BRD9 degradation inhibited the binding of RUNX2 at *Dmp1‐* and *Lef1*‐related intergenic regions, but had no influence in KLF4 recruitment. *** *p* < 0.001. ****p < 0.0001. ns (*p* > 0.05), not significant.
**Table S1:** Primer sequences for qRT‐PCR.
**Table S2:** Sequences for plasmid construction.
**Table S3:** Primer sequences for CUT&Tag‐qPCR.
**Table S4:** Expression level of RUNX2/KLF4‐interactors in cell populations from scRNA‐seq data (GSM5700359). (DM, dental mesenchyme; EP, epithelial cells; DF, dermal fibroblasts; Max_Cluster, cluster with the highest total expression; MaxAvg_Cluster, cluster with the highest average expression).

## Data Availability

The data that support the findings of this study are openly available in Genome Sequence Archive at https://ngdc.cncb.ac.cn/gsa [59, 60], reference number CRA036150.
