# Supplementary material for: Chromatin Remodeller BRD9 Orchestrates Odontoblastic Differentiation via Coordinating RUNX2‐KLF4
Source: Cell Prolif. 2026 Aug 6:e70269. Online ahead of print. doi: 10.1111/cpr.70269 (PMC13446069; doi:10.1111/cpr.70269)
Supplement: Supplementary file 1 [file CPR-9999-e70269-s001.doc]

Supplementary Materials

Chromatin Remodeler BRD9 Orchestrates Odontoblastic Differentiation via Coordinating RUNX2-KLF4

Wenrui Zeng^#1^, Yuxiu Lin^#1,2^, Yongyan Gao^1^, Delan Huang^1^, Yuanyuan Li^1,3^,

Honglei Ruan^1^, Guohua Yuan^1,4,5^, Huan Liu^*^^1,4,6^, Zhi Chen^*1,2^

1. State Key Laboratory of Oral & Maxillofacial Reconstruction and Regeneration, Key Laboratory of Oral Biomedicine Ministry of Education, Hubei Key Laboratory of Stomatology, School & Hospital of Stomatology, Wuhan University, Wuhan 430079, China.
2. Department of Cariology and Endodontics, School of Stomatology, Wuhan University, Wuhan 430079, China.
3. Xiamen Key Laboratory of Stomatological Disease Diagnosis and Treatment, Stomatological Hospital of Xiamen Medical College, Xiamen, China.
4. Frontier Science Center for Immunology and Metabolism, Wuhan University, China.
5. Hubei Provincial Key Laboratory of Developmentally Originated Disease, Wuhan 430071, China.
6. TaiKang Center for Life and Medical Sciences, Wuhan University, China.

**^#^Authors contributed equally to this work.**

^*^**Corresponding Authors:**

1. Zhi Chen: [zhichen@whu.edu.cn](mailto:zhichen@whu.edu.cn)
2. Huan Liu: [liu.huan@whu.edu.cn](mailto:liu.huan@whu.edu.cn)

Supplementary Figures

**Figure S1**

Figure S1. The transcriptomic profiling of scRNA-seq in E16.5 mouse molar characterized the expession of *Brd9*, *Runx2*, *Klf4* and signature markers. (A-C) Feature plot of *Brd9*, *Runx2* and *Klf4* in different cell populations. (D) Dotplot of signature genes in the cell populations.

**Figure S2**

Figure S2. Neural crest-specific ablation of *Brd9* results in cleft palate. (A-D) H&E staining for middle palate morphology at E15 and E17. *Brd9* cKO mice revealed a failure in palatal shelf fusion since E15. (E-J) H&E staining for palate morphology at PN0.5. *Brd9* cKO mice exhibited a complete cleft palate after birth. A, anterior palate; M, middle palate; P, posterior palate. Dashed lines referred to the midline where the palatal suture was located.

**Figure S3**

Figure S3. Expression pattern of BRD9, RUNX2 and KLF4 in mandibular first molars of *Wnt1*-Cre; *Brd9*^fl/fl^ and *Wnt1*-Cre; *Runx2*^fl/wt^; *Klf4*^fl/fl^ mice. (A-B) *Wnt1*-Cre; *Brd9*^fl/fl^ mice revealed distinct ablation of BRD9 in the dental papilla. (C-F) *Wnt1*-Cre; *Runx2*^fl/wt^; *Klf4*^fl/fl^ mice showed KLF4 ablation and RUNX2 downregulation in the dental mesenchyme. Od, odontoblasts; Am, ameloblasts. Representative immunopositive cells (brown DAB signals) are indicated by arrows.

**Figure S4**

Figure S4. Loss of *Brd9* influences early tooth development. (A-B) H&E staining for tooth germs of control and *Brd9* cKO mice at E15 and E17. DP, dental papilla. (C-D) Immunofluorescence of DSPP and DMP1 protein in molar germs. (E-F) Quantification of cusp height and normalized epithelial invagination depth in control and cKO tooth germs at E15 and E17. (G) Quantitative analysis of DMP1 and DSPP protein fluorescence intensity in dental papilla at E17. ***P* < 0.01. *** *P* < 0.001. ns (*p* > 0.05), not significant.

**Figure S5**

Figure S5. Loss of Brd9 does not affect early dental mesenchyme progenitor maintenance. (A) Immunofluorescence of SIX2, PAX9, Ki67 and CC-3 (Cleaved Caspase-3) proteins in the condensed dental mesenchyme at E12.5. DM, dental mesenchyme; DE, dental epithelium. (B) Quantitative analysis of the percentage of marker-positive area and marker-positive cell density (cells/100 μm²) in DM at E12.5. (C) Immunofluorescence of MSX1, PAX9, Ki67 and CC-3 in the dental papilla at E15. DP, dental papilla; EO, enamel organ. (D) Quantitative analysis of percentage of marker-positive area and marker-positive cell density (cells/100 μm²) in DP at E15. No significant differences were detected between the control and *Brd9* cKO embryos for the expression of above markers. ns (*p* > 0.05), not significant.

**Figure S6**

Figure S6. Nerual crest ablation of *Brd9* does not alter the expression of RUNX2 and KLF4 in the odontoblast layer. (A-B) Immunofluorescence of RUNX2 and KLF4 in molar cusps of control and *Brd9* cKO mice. (C-D) Quantitative analysis of DMP1 and DSPP protein fluorescence intensity. ns (*p* > 0.05), not significant.

**Figure S7**

Figure S7. BRD9 degradation does not affect RUNX2 or KLF4 expression during odontoblastic differentiation *in vitro*. (A) Western blot for RUNX2 and KLF4 expression at D5 of odontoblastic induction. (B) Quantitative analysis of RUNX2 and KLF4 protein levels. ns (*p* > 0.05), not significant.

**Figure S8**

Figure S8. Transcriptomic analysis for dBRD9-A treated mDPCs at Day 5. (A) Transcript levels of *Runx2* and *Klf4* at D5. ns (*p* > 0.05), not significant. (B) GO enrichment for upregulated genes upon BRD9 degradation. (C) KEGG pathway enrichment for upregulated genes. (D) KEGG pathway enrichment analysis revealed that TGF-β and Wnt signaling pathway were downregulated. (E) GSEA indicated significant downregulation of the extracellular matrix organization pathway. NES, Normalized Enrichment Score. (F) Heatmap of odontogenesis-related genes in NC and dB groups.

**Figure S9**

Figure S9. BRD9 maintains chromatin accessibility at odontogenic gene loci. (A-B) Visualization of ATAC-seq results at BRD9-dependent intergenic regions of *Dmp1* and *Lef1*. Data from two representative replicates per group were displayed. (C-D) CUT&Tag-qPCR results showed that BRD9 degradation inhibited the binding of RUNX2 at *Dmp1-* and *Lef1*-related intergenic regions, but had no influence in KLF4 recruitment. *** *P* < 0.001. *****P* < 0.0001. ns (*p* > 0.05), not significant.

**Supplementary Tables**

Table S1. Primer sequences for qRT-PCR

|  | Forward (5’ to 3’) | Reverse (5’ to 3’) |
| --- | --- | --- |
| *Dmp1* | ACCACAATACTGAATCTGAAAGCTC | TGCTGTCCGTGTGGTCACTA |
| *Alpl* | CCAACTCTTTTGTGCCAGAGA | GGCTACATTGGTGTTGAGCTTTT |
| *Brd9* | TTGGAGATGGAAGTCTGCTCT | GCAACTTGCTAGACAGTGAACT |
| *Runx2* | CACTGGCGGTGCAACAAGA | ATGACGGTAACCACAGTCCCATC |
| *Klf4* | GGGAAGTCGCTTCATGTGAGAG | GCGGGAAGGGAGAAGACACT |
| *Gapdh* | GAGAGTGTTTCCTCGTCCCG | ACTGTGCCGTTGAATTTGCC |

Table S2. Sequences for plasmid construction

A. Sequences of insert regions in plasmids

enhancer region of *Fam20c* ([chr5:138730843-138731272)](https://genome.ucsc.edu/cgi-bin/hgTracks?hgsid=3499343463_SijgRAPzqh1ILIUmEol0iN8LsT6a&db=mm10&position=chr5:138730843-138731273&hgPcrResult=pack)

ATTGTCCAGGAGATAGAAAGGAATGGGAGGAGATTGTGGCCCTTGAGGGGATGTGGTCTGTCAGCTGTATATGTTCTGCGAGCTAATGTAGCTTACCACCTTCGCCACTGTACATGACCCAAACTGGGCAGAGCCCACCACACCCCCTGAGTGACCTTTCCAGTCTCCTGGAAACTCCATCCTCAGTGAGGACACAACAACAGGAAGTGGCAGTTGGGTGGTTCCCTATGGTCTTATTTGCCTGGTATAAGAGGTCATTTCCTCAAACCACCCTCGTGTGGAAATTGTGGCTGTGCTGTGGTGATAGGAAGCATTTTTAGCCAATCAGCCTAGCCACCATGAACGAAAGGACGGGGAGAGGCAGGACATATTCATGTATCCACAGTAAAGGAGAACATGTTAGGAATGTAGAGACCTTCACACAGAGGCT

promoter region of *Fam20c* ([chr5:138754314-138754647](https://genome.ucsc.edu/cgi-bin/hgTracks?hgsid=3499343463_SijgRAPzqh1ILIUmEol0iN8LsT6a&db=mm10&position=chr5:138754314-138754647&hgPcrResult=pack))

GATTCCTACTTCCTACTAGGGGTTGGGGATCCAGAAGCCCTGTGGCCACAAATGAAGCCACGTGGTGAAAGCAGATCTCTTTTTACTGTGCACCCCGATTTAGCATCCCAGAGGGCAGGCAGCGAGACGCGCCCCGCCCACCGCCCGCGCATCCGCGCCACTCAGCCAGGCTCCGCGCGGCGCCGCCAGGGTTAAGGCGCAGGCGTGTCCCCTGGGGTGGGGTGGGCCCGGCAGCCTTGCCGAGCTTCTGGAGTCCCATCGGATCGGGGGCCCAACGCGCTCCTCCGCCGTTCTCTCCGCCGCGTGGCAACGTCGTCTCTTCGTCCTCCACCTA

B. Primers for plasmid construction (5’ to 3’)

| pGL3-Promoter plasmid | F-promoter | CCGGGCTCGAGATCTGCGATCGATTCCTACTTCCTACTAGGG |
| --- | --- | --- |
|  | R-promoter | CAGTACCGGAATGCCAAGCTTTAGGTGGAGGACGAAGAGACG |
|  | F-pGL3 | CGTCTCTTCGTCCTCCACCTAAAGCTTGGCATTCCGGTACTG |
|  | R-pGL3 | CCCTAGTAGGAAGTAGGAATCGATCGCAGATCTCGAGCCCGG |
| pGL3-Promoter-Enhancer plasmid | F1-enhancer | CATTTCTCTATCGATAGGTACATTGTCCAGGAGATAGAAAGG |
|  | R1-enhancer | CGAGCCCGGGCTAGCACGCGTAAGAGCTCGAGCCTCTGTGTGAAGGTCTC |
|  | F2-promoter | GTGCTAGCCCGGGCTCGAGATCTGCGATCGATTCCTACTTCCTACTAGGG |
|  | R2-promoter | CAGTACCGGAATGCCAAGCTTTAGGTGGAGGACGAAGAGACG |
|  | F3-pGL3 | CGTCTCTTCGTCCTCCACCTAAAGCTTGGCATTCCGGTACTG |
|  | R3-pGL3 | CCTTTCTATCTCCTGGACAATGTACCTATCGATAGAGAAATG |
| Pro-Enh-△motif1 | F-△1 | GGAGATTGTGGCCCTTGAGGGTCAGCTGTATATGTTCTGC |
|  | R-△1 | GCAGAACATATACAGCTGACCCTCAAGGGCCACAATCTCC |
| Pro-Enh-△motif2 | F-△2 | TGACCCAAACTGGGCAGAGCCCTGAGTGACCTTTCCAGTC |
|  | R-△2 | GACTGGAAAGGTCACTCAGGGCTCTGCCCAGTTTGGGTCA |
| Pro-Enh-△motif3 | F-△3 | GTATAAGAGGTCATTTCCTCTCGTGTGGAAATTGTGGCTG |
|  | R-△3 | CAGCCACAATTTCCACACGAGAGGAAATGACCTCTTATAC |

Table S3. Primer sequences for CUT&Tag-qPCR

|  | Forward (5’ to 3’) | Reverse (5’ to 3’) |
| --- | --- | --- |
| Dmp1-KLF4-P1 | CAGTGTCCAGTGCAGATATTAGC | ATGCAATGGGGACGGCTC |
| Dmp1-RUNX2-P1 | GCTCAGGAAGGACCATAGTTAGG | GCAGATTTAGAAAGAGGGGAGACA |
| Dmp1-RUNX2-P2 | CCGTCTATAGTGTCTCCCCTCT | TGTTCTGGGCCTTGCTATTCT |
| Lef1-  KLF4/RUNX2-P1 | CACGTGCCAACATCATCAGC | ATAAAGGGGTCCAGGGCTCT |
| Lef1-  KLF4/RUNX2-P2 | TGTCTTTCCAGTGTGCCTCA | TGCTCATTTTCCAAGCAGAAGC |
| Fam20c-  KLF4/RUNX2-P1 | CCAGGAGATAGAAAGGAATGGGA | GGCGAAGGTGGTAAGCTACA |
| Fam20c-  KLF4/RUNX2-P2 | TAGCTTACCACCTTCGCCAC | ACTGCCACTTCCTGTTGTTGT |
| Fam20c-  KLF4/RUNX2-P3 | GACACAACAACAGGAAGTGGC | ATGGTGGCTAGGCTGATTGG |
| Fam20c-KLF4-P4 | AGCATTTTTAGCCAATCAGCCT | CCTCTGTGTGAAGGTCTCTACA |

Table S4. Expression level of RUNX2/KLF4-interactors in cell populations from scRNA-seq data (GSM5700359).

(DM, dental mesenchyme; EP, epithelial cells; DF, dermal fibroblasts; Max_Cluster, cluster with the highest total expression; MaxAvg_Cluster, cluster with the highest average expression)

| Gene | Cluster0  (DM) | Cluster1  (EP) | Cluster2  (DF) | Max_  Cluster | MaxAvg_  Cluster |
| --- | --- | --- | --- | --- | --- |
| Rpl37 | 146899 | 148843 | 101460 | 1 | 7 |
| Rpsa | 138436 | 144895 | 92410 | 1 | 7 |
| Rpl11 | 129979 | 136167 | 93714 | 1 | 7 |
| Col3a1 | 99766 | 36374 | 132238 | 2 | 2 |
| Rpl23 | 115719 | 124468 | 78286 | 1 | 7 |
| Fth1 | 121316 | 75815 | 40356 | 0 | 4 |
| Rpl36 | 107977 | 117686 | 73129 | 1 | 7 |
| Rpl30 | 114393 | 111904 | 74447 | 0 | 7 |
| Rpl32 | 91477 | 110063 | 64949 | 1 | 7 |
| Rpl9 | 106166 | 106005 | 73756 | 0 | 7 |
| Rpl28 | 77894 | 91504 | 54368 | 1 | 7 |
| Rpl21 | 77043 | 85764 | 51207 | 1 | 7 |
| Gnas | 48669 | 52273 | 65953 | 2 | 6 |
| H3f3b | 59222 | 44336 | 25290 | 0 | 10 |
| H3f3a | 58063 | 51802 | 27291 | 0 | 0 |
| Rpl26 | 47615 | 55940 | 32474 | 1 | 7 |
| Gapdh | 31079 | 38487 | 17171 | 1 | 7 |
| Tpm1 | 38088 | 17806 | 11534 | 0 | 0 |
| Rpl17 | 30196 | 35894 | 21197 | 1 | 7 |
| Rpl13a | 27185 | 30547 | 16422 | 1 | 7 |
| Ppib | 28747 | 15749 | 26573 | 0 | 9 |
| S100a11 | 28136 | 28206 | 24673 | 1 | 8 |
| Rps25 | 23776 | 26740 | 14911 | 1 | 7 |
| Rps15 | 22746 | 25992 | 14273 | 1 | 7 |
| Eif5a | 24049 | 25755 | 13627 | 1 | 7 |
| Col6a1 | 14521 | 8684 | 25377 | 2 | 2 |
| Rpl5 | 22474 | 24543 | 14527 | 1 | 7 |
| Anxa2 | 17928 | 15853 | 22941 | 2 | 2 |
| Vcan | 22752 | 8562 | 11261 | 0 | 10 |
| Cfl1 | 22247 | 22130 | 11356 | 0 | 8 |
| Col5a2 | 20546 | 8117 | 18739 | 0 | 9 |
| Col5a1 | 12402 | 7635 | 19571 | 2 | 9 |
| Srsf3 | 19291 | 14433 | 7963 | 0 | 7 |
| Pdia6 | 18245 | 8053 | 9943 | 0 | 0 |
| Eif4g2 | 17117 | 14922 | 9599 | 0 | 10 |
| Fn1 | 15807 | 7781 | 16575 | 2 | 9 |
| Col6a3 | 13708 | 6902 | 16557 | 2 | 2 |
| Eif3f | 16461 | 15884 | 11273 | 0 | 7 |
| Arf5 | 16104 | 11878 | 12076 | 0 | 9 |
| Maged1 | 15938 | 9770 | 10781 | 0 | 9 |
| Prrx1 | 15844 | 7539 | 12351 | 0 | 2 |
| Col6a2 | 9962 | 6060 | 15466 | 2 | 2 |
| Itgb1 | 14377 | 7948 | 9899 | 0 | 8 |
| Serbp1 | 14293 | 14228 | 7080 | 0 | 7 |
| Rbm39 | 14204 | 8997 | 9325 | 0 | 10 |
| Hnrnpa1 | 13565 | 13898 | 5819 | 1 | 7 |
| Gnai2 | 13285 | 9601 | 7171 | 0 | 6 |
| Dnaja1 | 13253 | 10914 | 8385 | 0 | 6 |
| Cald1 | 12669 | 7531 | 6293 | 0 | 8 |
| Clic1 | 12433 | 10063 | 9644 | 0 | 11 |
| Capn6 | 4052 | 3480 | 12206 | 2 | 2 |
| Col14a1 | 2426 | 1120 | 11716 | 2 | 9 |
| Ahnak | 10647 | 7640 | 10667 | 2 | 2 |
| Tpi1 | 9233 | 10636 | 6959 | 1 | 1 |
| Flna | 10502 | 6694 | 5624 | 0 | 8 |
| Eif3i | 10379 | 10319 | 7509 | 0 | 7 |
| Gnb2 | 10337 | 10161 | 8092 | 0 | 8 |
| Pgam1 | 9074 | 10085 | 4807 | 1 | 4 |
| Prdx4 | 9980 | 6059 | 8439 | 0 | 9 |
| Akr1a1 | 9580 | 9965 | 7664 | 1 | 4 |
| Tmem59 | 9757 | 5058 | 6164 | 0 | 0 |
| Ywhaq | 9553 | 8600 | 5022 | 0 | 10 |
| Gsn | 6810 | 5436 | 9530 | 2 | 2 |
| Cnn2 | 9268 | 3591 | 2390 | 0 | 0 |
| Actc1 | 330 | 683 | 476 | 7 | 7 |
| Vcp | 9077 | 9011 | 5538 | 0 | 1 |
| Hdlbp | 8924 | 7777 | 7662 | 0 | 9 |
| Dstn | 8176 | 8807 | 4145 | 1 | 8 |
| Rhoc | 8804 | 7339 | 7505 | 0 | 6 |
| Hnrnpab | 8181 | 8728 | 3474 | 1 | 7 |
| Top2a | 7954 | 3306 | 694 | 0 | 0 |
| Lgals3 | 313 | 655 | 194 | 4 | 4 |
| Fbn1 | 6522 | 1239 | 7864 | 2 | 2 |
| Ddost | 7857 | 4651 | 6300 | 0 | 9 |
| Cct2 | 6862 | 7835 | 3760 | 1 | 7 |
| Csnk1a1 | 7664 | 7288 | 4336 | 0 | 10 |
| Glrx3 | 6534 | 7638 | 5171 | 1 | 7 |
| Hnrnpm | 7593 | 7153 | 3307 | 0 | 7 |
| Eif3e | 7119 | 7558 | 5188 | 1 | 1 |
| Ywhab | 7287 | 7363 | 4688 | 1 | 11 |
| Pcbp2 | 7133 | 7288 | 3987 | 1 | 1 |
| Cct6a | 6381 | 7276 | 3664 | 1 | 7 |
| Lrp1 | 7215 | 1483 | 6977 | 0 | 2 |
| Map1lc3b | 6542 | 7184 | 3945 | 1 | 6 |
| Ywhah | 6648 | 7160 | 4563 | 1 | 6 |
| Raly | 7096 | 6305 | 3219 | 0 | 6 |
| Pafah1b1 | 6989 | 6086 | 5013 | 0 | 2 |
| Capzb | 6746 | 6923 | 4187 | 1 | 4 |
| Top1 | 6901 | 6377 | 3668 | 0 | 7 |
| Ddb1 | 6615 | 6741 | 5126 | 1 | 2 |
| Sptbn1 | 6107 | 5497 | 6731 | 2 | 6 |
| Vdac2 | 6098 | 6727 | 3214 | 1 | 4 |
| Anxa6 | 6690 | 5614 | 5816 | 0 | 2 |
| Emilin1 | 6457 | 991 | 5229 | 0 | 9 |
| Eif3a | 6396 | 6196 | 4037 | 0 | 7 |
| Tpm3 | 5779 | 6312 | 3976 | 1 | 4 |
| U2af1 | 6210 | 5186 | 3194 | 0 | 7 |
| Fbln2 | 6185 | 927 | 5699 | 0 | 10 |
| Chd4 | 6164 | 2831 | 2811 | 0 | 10 |
| Sf3b2 | 6081 | 4576 | 3679 | 0 | 10 |
| Smarca5 | 5975 | 3656 | 3004 | 0 | 7 |
| Psmd7 | 5839 | 2364 | 3322 | 0 | 10 |
| Eif3c | 5575 | 5805 | 3644 | 1 | 11 |
| Sf3b1 | 5793 | 3769 | 3696 | 0 | 10 |
| Csde1 | 5757 | 4342 | 3421 | 0 | 10 |
| App | 5719 | 1510 | 4451 | 0 | 10 |
| Rer1 | 5611 | 1574 | 4472 | 0 | 10 |
| Hnrnpr | 5549 | 2499 | 2873 | 0 | 10 |
| Srsf5 | 5493 | 2037 | 3468 | 0 | 10 |
| Fbln1 | 5159 | 1553 | 5452 | 2 | 2 |
| Lman1 | 5166 | 1149 | 5418 | 2 | 2 |
| Atxn10 | 5322 | 2880 | 4032 | 0 | 2 |
| Syncrip | 5315 | 2697 | 2853 | 0 | 7 |
| St13 | 5261 | 2947 | 3968 | 0 | 2 |
| Srsf10 | 5239 | 2007 | 2775 | 0 | 7 |
| Snrnp70 | 5065 | 2203 | 2856 | 0 | 7 |
| Fkbp10 | 5044 | 1062 | 4342 | 0 | 2 |
| Csnk2a1 | 5031 | 2580 | 2862 | 0 | 10 |
| Matr3 | 5022 | 2050 | 3058 | 0 | 10 |
| Ubap2l | 4981 | 2084 | 3822 | 0 | 2 |
| Krt14 | 276 | 4978 | 216 | 1 | 1 |
| Rpn1 | 4974 | 1153 | 3972 | 0 | 11 |
| Kif5b | 4959 | 2992 | 2822 | 0 | 10 |
| Tardbp | 4906 | 2625 | 2733 | 0 | 7 |
| Psmc5 | 4884 | 2468 | 2786 | 0 | 7 |
| Ptbp1 | 4805 | 2632 | 2355 | 0 | 0 |
| Hspg2 | 2695 | 925 | 3516 | 6 | 10 |
| Impdh2 | 4760 | 2790 | 3111 | 0 | 7 |
| Rheb | 4755 | 2237 | 3046 | 0 | 8 |
| Prrc2b | 4689 | 1868 | 3125 | 0 | 0 |
| Tagln | 4659 | 1319 | 1452 | 0 | 8 |
| Thbs2 | 3669 | 723 | 4596 | 2 | 9 |
| Pcm1 | 4591 | 1731 | 2679 | 0 | 10 |
| Tra2b | 4587 | 1733 | 2340 | 0 | 9 |
| Ctnna1 | 4585 | 2289 | 2301 | 0 | 10 |
| Sec31a | 4476 | 1831 | 3847 | 0 | 2 |
| Hp1bp3 | 4403 | 1541 | 2286 | 0 | 9 |
| Eif3m | 4395 | 2579 | 2820 | 0 | 7 |
| Fkbp9 | 4372 | 1349 | 4191 | 0 | 2 |
| Psmc6 | 4371 | 2112 | 2580 | 0 | 7 |
| Hspa4 | 4366 | 2737 | 2042 | 0 | 7 |
| Dhx36 | 4353 | 1445 | 2112 | 0 | 0 |
| Suclg1 | 4342 | 2106 | 2908 | 0 | 7 |
| Actn1 | 4334 | 831 | 1217 | 0 | 8 |
| Usp9x | 4295 | 1955 | 2875 | 0 | 9 |
| Zc3h15 | 4293 | 2137 | 2654 | 0 | 10 |
| Copb2 | 4289 | 2213 | 4206 | 0 | 9 |
| Arpc5 | 3439 | 1870 | 2638 | 4 | 4 |
| Smc2 | 4268 | 1523 | 1065 | 0 | 10 |
| Efhd2 | 900 | 568 | 633 | 4 | 4 |
| Tnc | 4236 | 733 | 1476 | 0 | 10 |
| Mprip | 4215 | 1687 | 2688 | 0 | 8 |
| Tra2a | 4206 | 1082 | 2283 | 0 | 10 |
| Copa | 4099 | 1848 | 3842 | 0 | 9 |
| Psmc4 | 4085 | 2374 | 2135 | 0 | 6 |
| Eif3g | 4057 | 2322 | 2977 | 0 | 7 |
| Smc4 | 4047 | 1842 | 1115 | 0 | 7 |
| Copb1 | 4032 | 1207 | 3287 | 0 | 2 |
| Pa2g4 | 4028 | 2651 | 2253 | 0 | 7 |
| Pnn | 3993 | 1244 | 2165 | 0 | 7 |
| Parva | 3978 | 1405 | 2077 | 0 | 0 |
| Dnaja2 | 3963 | 2098 | 2572 | 0 | 7 |
| Rpn2 | 3955 | 1094 | 3310 | 0 | 9 |
| Igsf10 | 3953 | 500 | 2846 | 0 | 9 |
| Colgalt1 | 3085 | 810 | 3816 | 2 | 2 |
| Zfp207 | 3779 | 1846 | 2234 | 0 | 10 |
| Emilin2 | 1360 | 364 | 3776 | 2 | 11 |
| Msn | 1843 | 719 | 1570 | 6 | 6 |
| Ddr2 | 3760 | 851 | 2444 | 0 | 0 |
| Eif5b | 3760 | 1789 | 2784 | 0 | 11 |
| Alyref | 3722 | 1933 | 1721 | 0 | 7 |
| Gnai3 | 3712 | 1556 | 2538 | 0 | 8 |
| Dnajb6 | 3712 | 1961 | 2521 | 0 | 7 |
| Tkt | 3691 | 1692 | 2204 | 0 | 11 |
| Ppa1 | 3690 | 1891 | 2094 | 0 | 7 |
| Rsl1d1 | 3689 | 1606 | 2267 | 0 | 7 |
| Plxnb2 | 3679 | 1176 | 3208 | 0 | 8 |
| Nap1l4 | 3642 | 1967 | 2061 | 0 | 7 |
| Psmc2 | 3619 | 1842 | 2290 | 0 | 7 |
| Arcn1 | 3578 | 1565 | 3080 | 0 | 2 |
| Eif3d | 3553 | 2030 | 2290 | 0 | 7 |
| Psmd6 | 3551 | 1621 | 2436 | 0 | 7 |
| 9530068E07Rik | 3488 | 810 | 2601 | 0 | 10 |
| Trip12 | 3461 | 1643 | 2750 | 0 | 2 |
| Eif4e | 3440 | 1461 | 1451 | 0 | 7 |
| Rab10 | 3433 | 1382 | 2310 | 0 | 10 |
| Gpi1 | 2880 | 2377 | 2348 | 4 | 11 |
| Lpp | 3412 | 1187 | 2217 | 0 | 8 |
| Psmd13 | 3389 | 1533 | 1864 | 0 | 7 |
| Ckap5 | 3379 | 1343 | 1102 | 0 | 10 |
| Gja1 | 3371 | 1616 | 1945 | 0 | 0 |
| Nckap1 | 3364 | 1565 | 2160 | 0 | 8 |
| Xpo1 | 3353 | 1468 | 1672 | 0 | 7 |
| Strap | 3347 | 1657 | 2024 | 0 | 7 |
| Prc1 | 3344 | 966 | 247 | 0 | 10 |
| Ola1 | 3324 | 1513 | 1889 | 0 | 7 |
| Mcm7 | 3295 | 1660 | 1399 | 0 | 7 |
| Ilf3 | 3290 | 1234 | 1617 | 0 | 10 |
| Ppp2r1a | 3265 | 1937 | 2401 | 0 | 10 |
| Copg1 | 3233 | 1430 | 2898 | 0 | 9 |
| Gspt1 | 3229 | 1600 | 2197 | 0 | 7 |
| Stip1 | 3225 | 1937 | 1795 | 0 | 7 |
| Actn4 | 3210 | 1217 | 1931 | 0 | 6 |
| Jak1 | 3199 | 1045 | 2293 | 0 | 6 |
| Dhx15 | 3195 | 1486 | 1607 | 0 | 7 |
| Ppid | 3177 | 1584 | 2040 | 0 | 7 |
| Tcf3 | 3171 | 1638 | 2022 | 0 | 10 |
| Psmd1 | 3160 | 1705 | 1924 | 0 | 7 |
| Eif4a3 | 3156 | 1619 | 1870 | 0 | 7 |
| Rala | 3154 | 1542 | 2288 | 0 | 4 |
| Axl | 1896 | 496 | 3153 | 2 | 2 |
| Stt3b | 3144 | 659 | 2241 | 0 | 11 |
| Rcc2 | 3134 | 2172 | 959 | 0 | 10 |
| Psmd12 | 3132 | 1523 | 2011 | 0 | 7 |
| Pdia4 | 3118 | 686 | 1879 | 0 | 10 |
| Krt15 | 235 | 3106 | 138 | 1 | 1 |
| Rai14 | 3106 | 824 | 2015 | 0 | 0 |
| Eif2s1 | 3090 | 1634 | 1836 | 0 | 7 |
| Psmd11 | 3084 | 1465 | 1853 | 0 | 10 |
| Trip6 | 3060 | 1216 | 2670 | 0 | 2 |
| Ywhag | 3055 | 1760 | 2061 | 0 | 10 |
| Eif3l | 3050 | 1887 | 1962 | 0 | 7 |
| Eif3b | 2985 | 1799 | 1827 | 0 | 7 |
| Rad23b | 2976 | 1566 | 1991 | 0 | 9 |
| Cyfip1 | 2976 | 1344 | 1761 | 0 | 4 |
| Lims1 | 2956 | 1094 | 1930 | 0 | 8 |
| Thoc2 | 2897 | 1033 | 2160 | 0 | 2 |
| Tnrc6a | 2896 | 757 | 2162 | 0 | 10 |
| Rfc1 | 2886 | 1206 | 2300 | 0 | 2 |
| Gpc1 | 2870 | 657 | 808 | 0 | 7 |
| Ccn1 | 2857 | 130 | 1325 | 0 | 9 |
| Smarca1 | 2853 | 779 | 1609 | 0 | 0 |
| Psmc1 | 2848 | 1466 | 1674 | 0 | 7 |
| Ehd1 | 2030 | 943 | 2188 | 4 | 4 |
| Ap2a2 | 2837 | 1254 | 2625 | 0 | 4 |
| Fip1l1 | 2834 | 1018 | 1846 | 0 | 0 |
| Myo1b | 2830 | 754 | 811 | 0 | 8 |
| Rbm17 | 2812 | 1275 | 1928 | 0 | 7 |
| Timp3 | 2808 | 613 | 2233 | 0 | 10 |
| Rbbp6 | 2804 | 875 | 1689 | 0 | 10 |
| Emd | 2803 | 1149 | 1448 | 0 | 0 |
| Vcl | 2797 | 491 | 1130 | 0 | 8 |
| Aebp1 | 2782 | 369 | 2556 | 0 | 9 |
| Erbin | 2770 | 1157 | 1890 | 0 | 2 |
| Zfr | 2759 | 1085 | 2386 | 0 | 2 |
| Psmd3 | 2754 | 1277 | 1734 | 0 | 7 |
| Ahsa1 | 2754 | 1400 | 1995 | 0 | 7 |
| Mtch2 | 2753 | 1264 | 1860 | 0 | 7 |
| Abcf1 | 2746 | 1585 | 1686 | 0 | 7 |
| Mrps7 | 2734 | 1345 | 2058 | 0 | 7 |
| Asph | 2727 | 483 | 1724 | 0 | 11 |
| Atp2a2 | 2726 | 849 | 2465 | 0 | 8 |
| Ruvbl1 | 2713 | 1210 | 1614 | 0 | 7 |
| Arl2 | 2712 | 1313 | 1403 | 0 | 7 |
| Myef2 | 2705 | 948 | 1452 | 0 | 10 |
| Dock7 | 2700 | 825 | 1879 | 0 | 10 |
| U2af2 | 2681 | 1264 | 1430 | 0 | 7 |
| Luzp1 | 2681 | 936 | 1464 | 0 | 6 |
| Mrpl4 | 2659 | 1188 | 1380 | 0 | 4 |
| Col8a1 | 2656 | 255 | 666 | 0 | 0 |
| Hadha | 2630 | 875 | 1839 | 0 | 8 |
| Idh3b | 2628 | 1357 | 1906 | 0 | 7 |
| Snw1 | 2623 | 1034 | 1782 | 0 | 10 |
| Wdr1 | 2623 | 1230 | 1301 | 0 | 6 |
| Ipo5 | 2621 | 1376 | 1763 | 0 | 7 |
| Larp1 | 2609 | 1150 | 1733 | 0 | 7 |
| Dnmt1 | 2604 | 1068 | 1138 | 0 | 10 |
| Ik | 2602 | 1196 | 1701 | 0 | 4 |
| Immt | 2586 | 925 | 1371 | 0 | 7 |
| Pls3 | 2585 | 935 | 1920 | 0 | 9 |
| Vps35 | 2581 | 1174 | 2135 | 0 | 11 |
| Mrps34 | 2570 | 1443 | 1770 | 0 | 4 |
| Fasn | 2569 | 1252 | 2016 | 0 | 10 |
| Slc25a11 | 2561 | 1267 | 1769 | 0 | 11 |
| Wdr33 | 2529 | 817 | 1557 | 0 | 7 |
| Trp53 | 2521 | 1409 | 1175 | 0 | 7 |
| P4ha1 | 2435 | 459 | 2504 | 2 | 9 |
| Ahcyl1 | 2499 | 984 | 1618 | 0 | 0 |
| Rab5c | 2492 | 1428 | 1654 | 0 | 4 |
| Sh3gl1 | 2489 | 691 | 1933 | 0 | 9 |
| Snrnp200 | 2467 | 1133 | 1461 | 0 | 7 |
| Ap2b1 | 2447 | 1131 | 1733 | 0 | 10 |
| Ccdc80 | 1285 | 275 | 2446 | 2 | 9 |
| Fmr1 | 2443 | 823 | 1714 | 0 | 9 |
| Prpf19 | 2438 | 1146 | 1495 | 0 | 7 |
| P4ha2 | 1597 | 362 | 2431 | 2 | 2 |
| Ncor2 | 2429 | 1078 | 1989 | 0 | 11 |
| Sdha | 2403 | 925 | 1538 | 0 | 4 |
| Ganab | 2399 | 602 | 1597 | 0 | 10 |
| Hspb1 | 678 | 2350 | 1012 | 1 | 7 |
| Pfkl | 2349 | 1596 | 968 | 0 | 6 |
| Stt3a | 2336 | 601 | 2010 | 0 | 9 |
| Faf2 | 2334 | 372 | 1666 | 0 | 10 |
| Mcm4 | 2299 | 975 | 1427 | 0 | 7 |
| Hmgcs1 | 2294 | 1177 | 1642 | 0 | 8 |
| Zfp36l2 | 2287 | 879 | 1768 | 0 | 11 |
| Idh3g | 2267 | 1112 | 1371 | 0 | 4 |
| Epb41l3 | 1923 | 661 | 2242 | 2 | 10 |
| Gfpt1 | 2224 | 630 | 2064 | 0 | 9 |
| Ppp2r2a | 2220 | 1117 | 1761 | 0 | 10 |
| Nop56 | 2206 | 882 | 1146 | 0 | 7 |
| Zfp326 | 2202 | 587 | 1166 | 0 | 0 |
| Prkaca | 2197 | 1049 | 1520 | 0 | 7 |
| Mcm3 | 2194 | 1117 | 1231 | 0 | 7 |
| Slit3 | 2190 | 193 | 1349 | 0 | 0 |
| Cap1 | 1736 | 911 | 1308 | 4 | 4 |
| Brix1 | 2154 | 1129 | 1443 | 0 | 7 |
| Ppp1r12a | 2151 | 590 | 1284 | 0 | 8 |
| Fhl1 | 2151 | 474 | 1343 | 0 | 0 |
| Ranbp2 | 2150 | 814 | 1281 | 0 | 7 |
| Rangap1 | 2150 | 1010 | 559 | 0 | 10 |
| Dctn1 | 2148 | 1137 | 1773 | 0 | 10 |
| Poldip3 | 2127 | 984 | 1388 | 0 | 7 |
| Ipo7 | 2095 | 1087 | 1234 | 0 | 7 |
| Cttn | 2088 | 965 | 1684 | 0 | 10 |
| Sh3pxd2b | 2087 | 790 | 1830 | 0 | 10 |
| Magt1 | 2084 | 473 | 1551 | 0 | 10 |
| Ehd4 | 295 | 223 | 270 | 4 | 4 |
| Kif2a | 2067 | 836 | 1636 | 0 | 2 |
| Rbmx | 2050 | 551 | 798 | 0 | 10 |
| Cbfb | 2042 | 825 | 1055 | 0 | 0 |
| Hdac1 | 2037 | 898 | 1129 | 0 | 10 |
| Capn2 | 2031 | 921 | 1680 | 0 | 2 |
| Dclk1 | 2014 | 445 | 1984 | 0 | 9 |
| Mcm5 | 2012 | 918 | 892 | 0 | 7 |
| Tomm70a | 2010 | 969 | 1321 | 0 | 7 |
| Actr1b | 2009 | 979 | 1526 | 0 | 10 |
| Sec23a | 1983 | 706 | 1999 | 2 | 9 |
| Mark3 | 1998 | 725 | 1455 | 0 | 10 |
| Samm50 | 1986 | 1032 | 1213 | 0 | 4 |
| Lmo7 | 1982 | 249 | 352 | 0 | 0 |
| Adamts1 | 1453 | 187 | 1960 | 2 | 10 |
| Larp4 | 1948 | 699 | 1301 | 0 | 7 |
| Gtpbp4 | 1937 | 990 | 1093 | 0 | 7 |
| Itgav | 1916 | 434 | 1590 | 0 | 10 |
| Ap2a1 | 1912 | 735 | 1198 | 0 | 0 |
| Triobp | 1411 | 583 | 1907 | 2 | 2 |
| Farp1 | 1905 | 598 | 1677 | 0 | 8 |
| Usp5 | 1898 | 852 | 1155 | 0 | 0 |
| Racgap1 | 1873 | 636 | 205 | 0 | 10 |
| Xrcc1 | 1872 | 796 | 1455 | 0 | 7 |
| Tuba1c | 1620 | 594 | 246 | 4 | 4 |
| Brd9 | 1846 | 340 | 934 | 0 | 10 |
| Acly | 1836 | 843 | 1283 | 0 | 11 |
| Dbnl | 1832 | 911 | 1118 | 0 | 4 |
| Srp68 | 1820 | 817 | 1512 | 0 | 7 |
| Atxn2 | 1819 | 458 | 1244 | 0 | 10 |
| Snx4 | 1802 | 698 | 1478 | 0 | 2 |
| Tnpo1 | 1796 | 765 | 1435 | 0 | 7 |
| Cyp51 | 1786 | 324 | 1122 | 0 | 9 |
| Ddx18 | 1771 | 823 | 1231 | 0 | 7 |
| Tjp1 | 1522 | 457 | 1111 | 6 | 6 |
| Ppie | 1763 | 730 | 1022 | 0 | 7 |
| Wdr43 | 1763 | 698 | 1181 | 0 | 7 |
| Gcn1 | 1757 | 634 | 1298 | 0 | 2 |
| Fhl2 | 1756 | 378 | 910 | 0 | 8 |
| Ptbp3 | 1719 | 841 | 714 | 4 | 11 |
| Ecpas | 1736 | 657 | 1248 | 0 | 11 |
| Far1 | 1730 | 536 | 1195 | 0 | 10 |
| Ppp2r2d | 1727 | 821 | 1138 | 0 | 9 |
| Atl3 | 1725 | 318 | 1212 | 0 | 2 |
| Eif2s3x | 1719 | 955 | 847 | 0 | 7 |
| Fads2 | 1711 | 377 | 990 | 0 | 10 |
| Tns2 | 1711 | 319 | 1156 | 0 | 0 |
| Ncapd2 | 1710 | 636 | 285 | 0 | 7 |
| Cpne3 | 1707 | 762 | 1287 | 0 | 10 |
| Prkra | 1704 | 562 | 1176 | 0 | 9 |
| Csnk1e | 1703 | 727 | 1237 | 0 | 8 |
| Aldh18a1 | 1690 | 427 | 1357 | 0 | 9 |
| Decr1 | 1690 | 316 | 1067 | 0 | 11 |
| Rabgap1 | 1682 | 606 | 1307 | 0 | 2 |
| Abcd3 | 1682 | 366 | 1012 | 0 | 10 |
| Pycr2 | 1679 | 567 | 1161 | 0 | 7 |
| Arfgap1 | 1642 | 452 | 1670 | 2 | 9 |
| Xpnpep1 | 1669 | 661 | 1290 | 0 | 8 |
| Helz | 1669 | 386 | 1205 | 0 | 10 |
| Gart | 1662 | 881 | 1121 | 0 | 10 |
| Eftud2 | 1659 | 615 | 841 | 0 | 10 |
| Rbm7 | 1652 | 721 | 1029 | 0 | 10 |
| Sntb2 | 1645 | 360 | 1358 | 0 | 2 |
| Pdcd6ip | 1642 | 763 | 1378 | 0 | 4 |
| Specc1l | 1638 | 575 | 1102 | 0 | 0 |
| Frg1 | 1633 | 694 | 1147 | 0 | 11 |
| Stat3 | 1630 | 464 | 1271 | 0 | 10 |
| Lap3 | 1624 | 1164 | 1303 | 0 | 7 |
| Vasp | 1384 | 651 | 870 | 4 | 8 |
| Lbr | 1612 | 329 | 718 | 0 | 11 |
| Uhrf1 | 1604 | 906 | 663 | 0 | 7 |
| Ipo9 | 1603 | 483 | 937 | 0 | 0 |
| Dnm1l | 1603 | 570 | 1166 | 0 | 10 |
| Rpa1 | 1603 | 620 | 786 | 0 | 7 |
| Anln | 1601 | 310 | 140 | 0 | 10 |
| Dapk3 | 1588 | 731 | 1381 | 0 | 9 |
| Arhgap1 | 1583 | 538 | 1359 | 0 | 8 |
| Ogdh | 1581 | 602 | 1037 | 0 | 4 |
| Gigyf2 | 1580 | 501 | 1009 | 0 | 7 |
| Abcf2 | 1578 | 776 | 907 | 0 | 7 |
| Zfhx3 | 1577 | 631 | 1363 | 0 | 8 |
| Hadhb | 1577 | 615 | 1150 | 0 | 10 |
| Bms1 | 1553 | 552 | 983 | 0 | 7 |
| Cdc42bpb | 1543 | 618 | 1202 | 0 | 6 |
| Lonp1 | 1538 | 574 | 1015 | 0 | 7 |
| Ubr4 | 1527 | 442 | 1037 | 0 | 9 |
| Kpna3 | 1526 | 523 | 921 | 0 | 0 |
| Ddx3y | 1525 | 416 | 1463 | 0 | 2 |
| Mtrex | 1518 | 528 | 871 | 0 | 7 |
| Hsph1 | 1515 | 669 | 1309 | 0 | 7 |
| Pdxdc1 | 1506 | 494 | 1423 | 0 | 9 |
| Rc3h2 | 1504 | 369 | 1104 | 0 | 10 |
| Keap1 | 1502 | 522 | 1343 | 0 | 2 |
| Mical2 | 1498 | 124 | 109 | 0 | 0 |
| Plod3 | 1497 | 296 | 1318 | 0 | 9 |
| Zcchc8 | 1483 | 423 | 861 | 0 | 0 |
| Vps4b | 1482 | 599 | 1095 | 0 | 4 |
| Ddx21 | 1462 | 511 | 709 | 0 | 7 |
| Dkc1 | 1449 | 631 | 733 | 0 | 7 |
| Ythdf3 | 1446 | 431 | 970 | 0 | 0 |
| Ap3b1 | 1441 | 496 | 1148 | 0 | 2 |
| Mrpl3 | 1438 | 568 | 875 | 0 | 7 |
| Hspa14 | 1436 | 485 | 723 | 0 | 7 |
| Kif23 | 1435 | 451 | 94 | 0 | 11 |
| Esyt1 | 1346 | 172 | 1431 | 2 | 11 |
| Eif2a | 1426 | 520 | 976 | 0 | 7 |
| Afdn | 1426 | 481 | 1017 | 0 | 6 |
| Lemd2 | 1423 | 252 | 989 | 0 | 9 |
| Ppp2r5d | 1422 | 617 | 1004 | 0 | 7 |
| Slc25a24 | 1416 | 435 | 1197 | 0 | 2 |
| Lmf2 | 1412 | 265 | 1013 | 0 | 10 |
| Arf6 | 1409 | 570 | 1227 | 0 | 11 |
| Ppp1r9a | 980 | 242 | 1402 | 2 | 7 |
| Abhd16a | 1389 | 255 | 1277 | 0 | 2 |
| Ptpn9 | 1386 | 472 | 1134 | 0 | 8 |
| Ptprk | 1386 | 147 | 593 | 0 | 0 |
| Drg2 | 1382 | 553 | 916 | 0 | 7 |
| Itpr1 | 1375 | 210 | 1017 | 0 | 2 |
| Cpsf2 | 1371 | 493 | 837 | 0 | 10 |
| Tinagl1 | 83 | 38 | 101 | 6 | 6 |
| Ddx47 | 1367 | 463 | 928 | 0 | 10 |
| Arhgap35 | 1357 | 474 | 806 | 0 | 10 |
| Dnm2 | 1356 | 441 | 838 | 0 | 4 |
| Myof | 1353 | 351 | 949 | 0 | 8 |
| Cpsf3 | 1346 | 549 | 932 | 0 | 7 |
| Casc3 | 1342 | 382 | 913 | 0 | 10 |
| Tufm | 1313 | 527 | 857 | 0 | 7 |
| Usp4 | 1310 | 449 | 880 | 0 | 10 |
| Ppp1r12c | 1286 | 376 | 1310 | 2 | 8 |
| Dnajc13 | 1308 | 369 | 955 | 0 | 9 |
| Smad2 | 1304 | 420 | 859 | 0 | 10 |
| Myo18a | 1297 | 391 | 612 | 0 | 10 |
| Tmx3 | 1297 | 220 | 836 | 0 | 10 |
| Klc1 | 1258 | 373 | 1017 | 0 | 2 |
| Rfc5 | 1258 | 496 | 583 | 0 | 11 |
| Nxf1 | 1252 | 395 | 774 | 0 | 10 |
| Uggt1 | 1249 | 234 | 998 | 0 | 2 |
| Mrps9 | 1232 | 455 | 806 | 0 | 7 |
| Ddx27 | 1232 | 441 | 696 | 0 | 7 |
| Upf3b | 1221 | 381 | 358 | 0 | 10 |
| Atad3a | 1203 | 439 | 794 | 0 | 7 |
| Arhgef2 | 1200 | 524 | 957 | 0 | 7 |
| Araf | 1198 | 256 | 915 | 0 | 9 |
| Ap3d1 | 1195 | 344 | 1011 | 0 | 9 |
| Uba6 | 1190 | 409 | 773 | 0 | 9 |
| Pdcd11 | 1180 | 404 | 738 | 0 | 7 |
| Tdrd3 | 1177 | 318 | 795 | 0 | 9 |
| Sec24b | 1173 | 371 | 847 | 0 | 2 |
| Elp1 | 1168 | 402 | 862 | 0 | 7 |
| Lactb | 1164 | 166 | 537 | 0 | 4 |
| Smtn | 1164 | 265 | 985 | 0 | 8 |
| Crnkl1 | 1161 | 359 | 553 | 0 | 7 |
| Upf2 | 1160 | 352 | 839 | 0 | 9 |
| Snx9 | 1159 | 389 | 1102 | 0 | 2 |
| Lrrfip2 | 1155 | 484 | 655 | 0 | 9 |
| Ncapg | 1147 | 431 | 87 | 0 | 7 |
| Diaph3 | 1143 | 206 | 188 | 0 | 10 |
| Pdk3 | 1138 | 316 | 537 | 0 | 0 |
| Cse1l | 1125 | 486 | 447 | 0 | 7 |
| Myo9b | 1122 | 354 | 1029 | 0 | 9 |
| Zfp703 | 1079 | 498 | 1120 | 2 | 9 |
| Tbc1d17 | 1117 | 349 | 1109 | 0 | 9 |
| Ltbp2 | 960 | 75 | 1117 | 2 | 2 |
| Dcaf7 | 1116 | 457 | 659 | 0 | 11 |
| Ccar2 | 1113 | 342 | 668 | 0 | 0 |
| Mrps30 | 1110 | 409 | 823 | 0 | 7 |
| Xpo5 | 1099 | 377 | 660 | 0 | 10 |
| Esyt2 | 1092 | 207 | 951 | 0 | 11 |
| Rcc1 | 1090 | 303 | 374 | 0 | 7 |
| Dlat | 1080 | 417 | 793 | 0 | 7 |
| Wdr5 | 1076 | 408 | 698 | 0 | 7 |
| Kif4 | 1064 | 338 | 129 | 0 | 10 |
| Ankfy1 | 1062 | 301 | 769 | 0 | 11 |
| Nub1 | 1057 | 368 | 839 | 0 | 8 |
| Lrpprc | 1056 | 386 | 683 | 0 | 7 |
| Sacm1l | 1056 | 279 | 796 | 0 | 2 |
| Trim32 | 1054 | 297 | 685 | 0 | 10 |
| Pef1 | 1050 | 416 | 771 | 0 | 10 |
| Ipo4 | 922 | 329 | 1049 | 2 | 2 |
| Yes1 | 1047 | 199 | 590 | 0 | 6 |
| Unc45a | 1042 | 356 | 742 | 0 | 8 |
| Plrg1 | 1032 | 394 | 632 | 0 | 7 |
| Dync1li1 | 1028 | 321 | 724 | 0 | 7 |
| Otud4 | 1014 | 339 | 763 | 0 | 7 |
| Nob1 | 1013 | 279 | 613 | 0 | 7 |
| Dcaf13 | 1003 | 366 | 638 | 0 | 7 |
| Vrk3 | 997 | 341 | 643 | 0 | 10 |
| Camk2g | 994 | 292 | 664 | 0 | 8 |
| Cyp20a1 | 877 | 175 | 985 | 2 | 10 |
| Xrcc6 | 984 | 390 | 546 | 0 | 7 |
| Cpsf1 | 979 | 366 | 582 | 0 | 7 |
| Ptx3 | 979 | 64 | 325 | 0 | 0 |
| Plxna1 | 967 | 225 | 762 | 0 | 8 |
| Top3b | 950 | 292 | 729 | 0 | 9 |
| Myo1e | 942 | 215 | 194 | 0 | 0 |
| Psmd5 | 933 | 386 | 565 | 0 | 0 |
| Kpna1 | 915 | 252 | 524 | 0 | 11 |
| Por | 913 | 181 | 851 | 0 | 11 |
| Xrcc5 | 907 | 392 | 451 | 0 | 7 |
| Dvl3 | 905 | 263 | 728 | 0 | 2 |
| Apbb1ip | 786 | 117 | 711 | 4 | 4 |
| Pes1 | 902 | 380 | 601 | 0 | 7 |
| Zfp608 | 899 | 167 | 655 | 0 | 10 |
| Fmnl3 | 895 | 223 | 412 | 0 | 6 |
| Ripk3 | 894 | 210 | 448 | 0 | 11 |
| Zc2hc1a | 875 | 356 | 636 | 0 | 8 |
| Slu7 | 871 | 261 | 628 | 0 | 10 |
| Nol6 | 870 | 310 | 581 | 0 | 7 |
| Slc30a7 | 869 | 114 | 726 | 0 | 9 |
| Tmem43 | 864 | 158 | 781 | 0 | 2 |
| Usp39 | 859 | 332 | 407 | 0 | 10 |
| Nsf | 834 | 206 | 552 | 0 | 4 |
| Nop2 | 823 | 302 | 532 | 0 | 7 |
| Stn1 | 805 | 228 | 548 | 0 | 9 |
| Prkci | 804 | 215 | 463 | 0 | 0 |
| Rasa3 | 804 | 301 | 339 | 0 | 10 |
| Slc25a12 | 799 | 309 | 723 | 0 | 2 |
| Atf6 | 789 | 125 | 528 | 0 | 10 |
| Cip2a | 775 | 178 | 110 | 0 | 10 |
| Kpna2 | 774 | 219 | 85 | 0 | 7 |
| Peak1 | 760 | 145 | 673 | 0 | 8 |
| Rpe | 753 | 231 | 445 | 0 | 10 |
| Trip13 | 751 | 166 | 123 | 0 | 7 |
| Tfrc | 606 | 255 | 204 | 4 | 4 |
| Smurf1 | 736 | 227 | 508 | 0 | 10 |
| Trim25 | 417 | 234 | 331 | 6 | 6 |
| Srbd1 | 685 | 260 | 431 | 0 | 10 |
| Iqgap3 | 673 | 70 | 56 | 0 | 10 |
| Hyou1 | 670 | 116 | 388 | 0 | 10 |
| Fkbp15 | 669 | 158 | 447 | 0 | 4 |
| Agk | 669 | 80 | 465 | 0 | 7 |
| Rab3gap2 | 665 | 167 | 583 | 0 | 11 |
| Mink1 | 663 | 242 | 540 | 0 | 10 |
| Nup160 | 659 | 138 | 363 | 0 | 7 |
| Ttll12 | 657 | 196 | 424 | 0 | 7 |
| Eif2ak2 | 648 | 76 | 334 | 0 | 0 |
| Sema4c | 637 | 97 | 368 | 0 | 10 |
| Rab2b | 636 | 84 | 437 | 0 | 9 |
| Mrps27 | 629 | 217 | 510 | 0 | 7 |
| Rrp9 | 627 | 296 | 356 | 0 | 7 |
| Actg2 | 173 | 36 | 20 | 8 | 8 |
| Nfs1 | 599 | 254 | 430 | 0 | 7 |
| Fhod1 | 597 | 53 | 351 | 0 | 8 |
| Inf2 | 471 | 175 | 591 | 2 | 10 |
| Nubpl | 591 | 138 | 277 | 0 | 10 |
| Trim56 | 573 | 122 | 362 | 0 | 10 |
| Tom1l2 | 568 | 118 | 435 | 0 | 10 |
| Apobec3 | 179 | 29 | 107 | 4 | 11 |
| Zc3hav1 | 230 | 55 | 212 | 4 | 11 |
| Ddx20 | 544 | 126 | 306 | 0 | 10 |
| Heatr1 | 543 | 155 | 240 | 0 | 7 |
| Nat10 | 542 | 124 | 344 | 0 | 7 |
| Slc27a4 | 530 | 76 | 423 | 0 | 10 |
| Aacs | 510 | 132 | 501 | 0 | 8 |
| Isg15 | 177 | 12 | 44 | 4 | 4 |
| Psat1 | 501 | 356 | 248 | 0 | 7 |
| Slc35b3 | 487 | 68 | 372 | 0 | 2 |
| Zfp609 | 476 | 95 | 329 | 0 | 9 |
| Pisd | 464 | 118 | 365 | 0 | 10 |
| Hsd17b11 | 461 | 35 | 414 | 0 | 11 |
| Cdk17 | 453 | 102 | 151 | 0 | 6 |
| Poc1b | 448 | 106 | 243 | 0 | 7 |
| Itpr3 | 191 | 140 | 212 | 6 | 10 |
| Ldlr | 432 | 76 | 316 | 0 | 11 |
| Sun2 | 430 | 45 | 120 | 0 | 10 |
| Gfpt2 | 200 | 26 | 401 | 2 | 2 |
| Cep55 | 400 | 82 | 33 | 0 | 10 |
| Nexn | 400 | 87 | 313 | 0 | 7 |
| Myo7a | 142 | 26 | 70 | 4 | 4 |
| Rras2 | 375 | 122 | 199 | 0 | 7 |
| Tuba4a | 85 | 97 | 42 | 4 | 11 |
| Nup107 | 372 | 79 | 189 | 0 | 7 |
| Plekhg3 | 363 | 112 | 344 | 0 | 10 |
| Ephx1 | 186 | 41 | 352 | 2 | 2 |
| Steap3 | 347 | 17 | 97 | 0 | 0 |
| Acsl1 | 290 | 73 | 180 | 4 | 7 |
| Coro2b | 231 | 61 | 189 | 6 | 10 |
| Kif1a | 322 | 144 | 154 | 0 | 10 |
| Pfkp | 274 | 112 | 178 | 4 | 11 |
| Pdlim5 | 281 | 121 | 121 | 6 | 6 |
| Parp12 | 310 | 42 | 237 | 0 | 4 |
| Lss | 298 | 65 | 188 | 0 | 11 |
| Acsl4 | 294 | 82 | 212 | 0 | 10 |
| Cpeb4 | 228 | 50 | 217 | 4 | 4 |
| Dhx37 | 266 | 79 | 144 | 0 | 11 |
| Epha2 | 249 | 54 | 40 | 0 | 10 |
| Rrp12 | 249 | 75 | 171 | 0 | 7 |
| Ston1 | 237 | 29 | 134 | 0 | 10 |
| Krt19 | 19 | 218 | 24 | 1 | 1 |
| Rbm45 | 209 | 50 | 136 | 0 | 11 |
| Ifi202b | 161 | 42 | 26 | 4 | 4 |
| Irgm1 | 186 | 23 | 76 | 0 | 6 |
| Kifc5b | 162 | 29 | 19 | 0 | 9 |
| Serpind1 | 6 | 2 | 7 | 4 | 4 |
| Pcdhb17 | 145 | 18 | 103 | 0 | 9 |
| Helz2 | 123 | 31 | 114 | 0 | 11 |
| Hspa1b | 120 | 98 | 110 | 0 | 8 |
| Frmd3 | 117 | 22 | 71 | 0 | 7 |
| Serpine1 | 95 | 7 | 81 | 0 | 8 |
| Abcb1b | 57 | 3 | 40 | 4 | 9 |
| Vtn | 5 | 1 | 7 | 8 | 8 |
| Anxa8 | 4 | 28 | 1 | 1 | 1 |
| Abcg8 | 17 | 0 | 0 | 0 | 0 |
| Serpinb12 | 0 | 16 | 0 | 1 | 1 |
| Pkp2 | 0 | 0 | 0 | 4 | 4 |
| Diras2 | 4 | 11 | 1 | 1 | 1 |
| Aldh1l2 | 9 | 0 | 3 | 0 | 10 |
| Krt75 | 0 | 7 | 1 | 1 | 1 |
| F5 | 1 | 0 | 0 | 4 | 4 |
| Loxl4 | 3 | 0 | 0 | 0 | 0 |
| Serpinc1 | 0 | 0 | 0 | 3 | 4 |
| Ciita | 1 | 0 | 0 | 0 | 0 |
| Cct6b | 1 | 1 | 0 | 0 | 1 |
| Cpb2 | 0 | 0 | 0 | 0 | 0 |
| Fgg | 0 | 0 | 0 | 0 | 0 |
| Gemin4 | 0 | 0 | 0 | 0 | 0 |
| Hc | 0 | 0 | 0 | 0 | 0 |
| A2m | 0 | 0 | 0 | 0 | 0 |
| Onecut2 | 0 | 0 | 0 | 0 | 0 |
| Plet1 | 0 | 0 | 0 | 0 | 0 |
| Rgn | 0 | 0 | 0 | 0 | 0 |
